# *Plasmodium* Protease ROM1 Is Important for Proper Formation of the Parasitophorous Vacuole

**DOI:** 10.1371/journal.ppat.1002197

**Published:** 2011-09-01

**Authors:** Iset Medina Vera, Wandy L. Beatty, Photini Sinnis, Kami Kim

**Affiliations:** 1 Departments of Medicine and of Microbiology and Immunology, Albert Einstein College of Medicine, Bronx, New York, United States of America; 2 Department of Molecular Microbiology, Washington University School of Medicine, St. Louis, Missouri, United States of America; 3 Department of Microbiology, New York University Langone School of Medicine, New York, New York, United States of America; Weill Medical College of Cornell University, United States of America

## Abstract

Apicomplexans are obligate intracellular parasites that invade host cells by an active process leading to the formation of a non-fusogenic parasitophorous vacuole (PV) where the parasite replicates within the host cell. The rhomboid family of proteases cleaves substrates within their transmembrane domains and has been implicated in the invasion process. Although its exact function is unknown, *Plasmodium* ROM1 is hypothesized to play a role during invasion based on its microneme localization and its ability to cleave essential invasion adhesins. Using the rodent malaria model, *Plasmodium yoelii*, we carried out detailed quantitative analysis of *pyrom1* deficient parasites during the *Plasmodium* lifecycle. *Pyrom1(-)* parasites are attenuated during erythrocytic and hepatic stages but progress normally through the mosquito vector with normal counts of oocyst and salivary gland sporozoites. *Pyrom1* steady state mRNA levels are upregulated 20-fold in salivary gland sporozoites compared to blood stages. We show that *pyrom1(-)* sporozoites are capable of gliding motility and traversing host cells normally. Wildtype and *pyrom1(-)* sporozoites do not differ in the rate of entry into Hepa1–6 hepatocytes. Within the first twelve hours of hepatic development, however, only 50% *pyrom1(-)* parasites have developed into exoerythrocytic forms. Immunofluorescence microscopy using the PVM marker UIS4 and transmission electron microscopy reveal that the PV of a significant fraction of *pyrom1(-)* parasites are morphologically aberrant shortly after invasion. We propose a novel function for PyROM1 as a protease that promotes proper PV modification to allow parasite development and replication in a suitable environment within the mammalian host.

## Author Summary

*Plasmodium* parasites are obligate intracellular organisms that invade cells by an active mechanism mediated by the secretion of contents from specialized secretory organelles, the micronemes and rhoptries. Invaded parasites reside and replicate within a membrane-bound compartment called the parasitophorous vacuole (PV). PV formation is exclusive to development within mammalian specific host cells, the erythrocytes and hepatocytes. Proper modification of the PV is important to protect the parasite from host defenses and to serve as a gateway for nutrient acquisition and communication with the environment. The rhomboid proteins, a class of intramembrane serine proteases, are implicated in the invasion process. We studied the microneme rhomboid protease, ROM1 in the rodent malaria parasite, *Plasmodium yoelii*. We find that pyROM1 is not important for efficient invasion into host cells and instead is important for survival within the host cells. Analysis of parasites developing within hepatocytes reveals a defect in PV development. We propose that pyROM1 provides a fitness advantage to parasites developing within host cells by promoting the proper modification of the PV.

## Introduction

Malaria is a pervasive infectious disease that causes one million deaths each year and exacerbates the social and economic instability of endemic areas [1]. Malaria is caused by *Plasmodium* species, obligate intracellular protozoan parasites in the phylum Apicomplexa. *Plasmodium spp* have a complex life cycle with multiple differentiated forms that cycle between a sexual stage in the mosquito vector and an asexual stage in the vertebrate host. As obligate intracellular parasites, apicomplexans invade cells through the use of highly specialized secretory organelles, the micronemes and rhoptries [2]. Secretion from the micronemes is concurrent with apical reorientation and attachment of the parasite to the host cell membrane [3]. Tight apposition between the parasite and host cell plasma membrane forms the moving junction through the cooperation of the microneme adhesin AMA1 and the Rhoptry Neck proteins (RON) [4]–[6]. As the parasite pushes itself forward into the host cell, the moving junction, a constrictive ring that translocates posteriorly and forms a parasitophorous vacuole (PV) forms through the invagination of host cell plasma membrane. PV formation is accompanied by the secretion of rhoptry contents in the form of secretory vesicles that become incorporated into the nascent parasitophorous vacuole membrane (PVM) [7]–[9]. The PV is devoid of most host cell proteins and avoids fusion with host lysosomes [10]–[11]. Proper establishment and modification of the nascent PV is critical for the survival of the parasite.

Sporozoites and merozoites are the invasive stages of *Plasmodium* parasites that form a PV as they enter their host cells, the hepatocytes and the red blood cells (RBC), respectively. Until recently, studies of parasite invasion and development in hepatocytes have been limited. Much of what we know about apicomplexan host cell invasion and PV formation comes from studies using the model apicomplexan, *Toxoplasma gondii*, and from studies using *Plasmodium falciparum* erythrocytic stages [2], [12]. It is likely, however, that sporozoites invade host cells in a manner similar to *T. gondii* tachyzoites as illustrated in a recent study showing the importance of host F-actin polymerization at the site of parasite entry for *T. gondii* tachyzoites and *Plasmodium* sporozoites [13].

Transcriptomic and proteomic analysis of *Plasmodium* sporozoites have enabled a gene-based approach to studying this important stage [14]–[17]. Sporozoites developing within the hepatocyte undergo a radical transformation within the first few hours post invasion. The intracellular sporozoite within its PV settles near the nucleus of host cells and modifies its long and polarized shape to become spherical [18]–[19]. The liver stage parasite grows and replicates within the PV ultimately releasing membrane bound bundles of thousands of daughter merozoites (merosomes) that then enter the erythrocytic cycle [20]–[21].

Pre-erythrocytic stages are considered targets for the development of vaccines and prophylactic drugs [22]–[23]. Recently, genetically modified live attenuated sporozoites that confer sterile immunity in rodent models were generated. The deleted genes, UIS3, UIS4, and P52 are upregulated in salivary gland sporozoites and are crucial for development within hepatocytes. Their gene products localize to the micronemes/secretory organelles of salivary gland sporozoites and are necessary for early liver stage development [24]–[26].

Host cell invasion by apicomplexans is associated with proteolysis of surface proteins, which include resident surface antigens and apically-secreted adhesion molecules, collectively called adhesins [27]–[30]. Proteases implicated in shedding of adhesins are parasite-encoded and include the subtilase class of serine proteases and the rhomboid class of serine proteases [31]. Rhomboid proteins are polytopic membrane-associated serine proteases that form a catalytic pocket within the lipid bilayer and have the unique characteristic of cleaving substrates within the transmembrane domain [32]–[34]. Rhomboid proteases recognize small residues such as glycine and alanine within the transmembrane domains of substrates [35]. In the Apicomplexa, processing of key microneme adhesins, such as TgMIC2, TgMIC6, TgAMA1 and PfEBA175, has been shown to occur via intramembranous cleavage at sites predicted to be rhomboid-like substrates [36]–[38], [29].

The specific rhomboid protease(s) involved in these processes have not been identified. Based on expression, localization, and substrate specificity, two *Plasmodium* rhomboids, ROM1 and ROM4, are predicted to play a role during invasion. PfROM1 is co-localizes with microneme markers has the ability to cleave certain microneme invasion adhesins such as PfAMA1, PfMAEBL, certain EBLs, and rhoptry proteins of the Rh family in a cell based assay [29], [39]. In addition, a separate study reported that PfROM1 is localized to a new apical organelle, the mononeme [40]. When expression of the PfROM1 ortholog in *Toxoplasma gondii*, TgROM1, is down regulated in tachyzoite parasites, there is a slight growth phenotype and mild invasion defect compared to the wildtype control [41]. Disruption of *pbrom1* in *Plasmodium berghei* led to attenuation ascribed to an invasion defect [42], however, invasion of parasites into host cells was not directly tested in this study.

It is still unclear if ROM1 plays a role during *Plasmodium* parasite invasion. Using the rodent malaria parasite, *Plasmodium yoelii*, we investigated the specific role of ROM1 during the parasite lifecycle. We show that ROM1 is not required for entry into host cells but, instead, ROM1 is necessary shortly after the parasite has entered the host cell to promote fitness and parasite survival.

## Results

### PyROM1 is expressed in all invasive stages of the parasite lifecycle, with increased expression during sporozoite stages

*Pyrom1* was cloned from cDNA of *Plasmodium yoelii* 17XNL mixed blood stages using 5′ and 3′ Rapid Amplification of cDNA Ends (Smart RACE Clontech). The sequence obtained consists of 4 exons and 3 introns, encompassing two annotated genes on PlasmoDB, py00729 and py00728. Based on topology predictions (TMHMM and HMMTOP), PyROM1 has seven transmembrane domains with the canonical rhomboid catalytic serine motif (GASTS) found within transmembrane domain four and a conserved histidine found within transmembrane domain six (merops.sanger.ac.uk). It has an N-terminal tail of 52 amino acids that includes the conserved microneme targeting motif YPHY [43] and a very short carboxy terminal tail (Figure S1).

**Figure 1:**
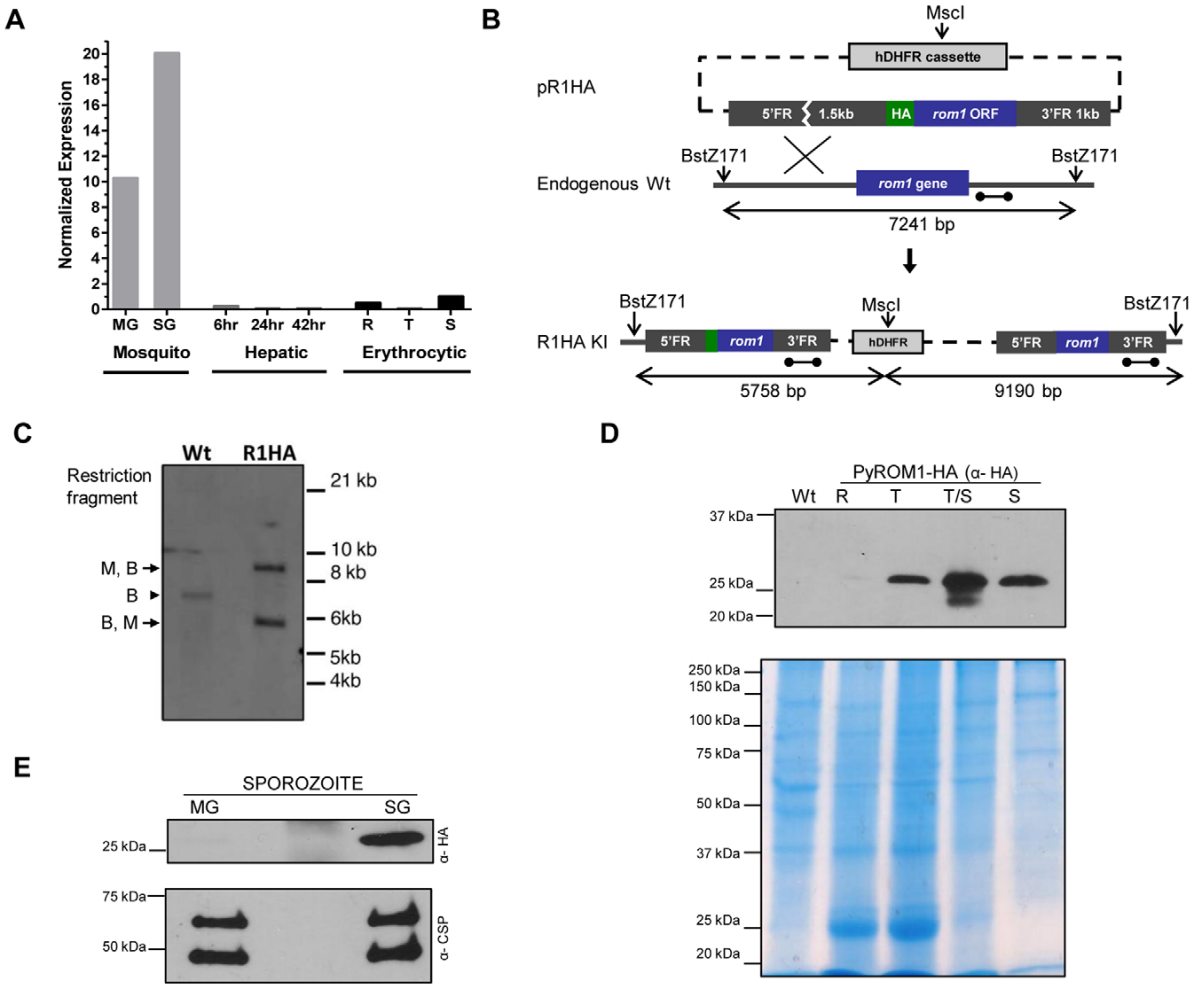
Stage-specific expression of *pyROM1* during the malaria life cycle. (A) Real time PCR using cDNA prepared from synchronized blood stage parasites, mosquito derived parasites, and hepatic stage parasites of *P. yoelii* 17XNL. Expression levels were normalized to 18s rRNA and calculated relative to the Schizont (S) transcripts, set as 1. Parasite stages are abbreviated as follows: MG, Midgut sporozoites (Day 10); SG, Salivary Gland sporozoites (Day 14); 6, sporozoite development at 6 hours post-invasion; 24, Liver trophozoite development at 24 hours; 42, Liver schizont development at 42 hours; R, Blood stage rings; T, blood stage trophozoite; S, blood stage schizont. (B) Schematic of knock-in strategy using pR1HA construct consisting of the *pyrom1* ORF fused to an N-terminal triple HA tag under the control of its 5′ and 3′ regulatory elements. (C) Restriction digest strategy described in (B) was used to test integration of the knock-in construct. Homologous recombination of knock-in construct pR1HA into the *pyrom1* locus creates an MscI (M) restriction site that is specific to the hDHFR coding sequence. Restriction sites BstZ171 (B) lie just outside the *pyrom1* sequence used in pR1HA knock in vector. Digestion of PyROM1-HA (R1HA) gDNA yields a two fragment restriction pattern (B,M – 5.758 bp; M,B – 9.190 bp) when a probe specific to the 3′ flanking region is used (dumbbell bar). Digestion of wildtype (Wt) gDNA with both enzymes yields a single fragment (B,B – 7241 bp) using the same probe. (D) Western blot analysis of synchronized blood stage parasites using lysates from pyROM1-HA recombinant clone probed with anti-HA antibody reveals a band of the expected size, ∼30 kDa (R: Ring, T: Trophozoite, T/S: late Trophozoite/Schizont, S: Schizont). Mixed blood stages of wildtype parasites were loaded as a negative control (Wt). Saponin-released parasite lysates were loaded at 40 ug per lane to normalize for protein amounts. Coomassie Blue stained gel is shown below Western blot to serve as a loading control (E) Western blot analysis of midgut (MG) and salivary gland (SG) sporozoites from pyROM1-HA knock in parasites probed with anti-HA antibody (100,000 sporozoites/lane). Shown below is the same Western blot probed with anti-PyCSP (2F6) antibody to serve as loading control.

Based on the *P. falciparum* DNA microarray data, expression of *pfrom1* is similar to genes involved during invasion with a significant upregulation in the sporozoite stage [44]–[45]. We quantified *pyrom1* mRNA in several stages of the malaria life cycle using quantitative RT-PCR (Figure 1A). Amplification of *Pyrom1* cDNA from synchronized erythrocytic stages shows modest expression, with greatest expression in schizont (S) stages. There is a 10-fold increased expression in midgut (MG) sporozoites and a 20-fold increased expression in salivary gland (SG) sporozoites relative to expression levels of schizont stages. To control for appropriate expression analysis, additional expression profiles of other known genes (PyAMA1, PyUIS3, PyADA, PyTUB1, and PyCSP) was carried out using the same cDNA (Figure S2).

To further characterize pyROM1 expression at the protein level, we generated a transgenic line that expresses pyROM1 tagged at the N-terminus with a triple hemaglutinin tag (3xHA). Single cross-over homologous recombination using hDHFR selection cassette yields the expression of the HA-tagged *pyrom1* open reading frame driven by its own 5′ and 3′ regulatory elements (Figure 1B). Successful integration of the knock-in construct was verified by Southern blot analysis of genomic DNA from mixed erythrocytic stage transgenic parasites R1HA (Figure 1C). Western blot analysis of erythrocytic and sporozoite stages of PyROM1-HA reveals a band that migrates at the expected size of ∼30 kDa on SDS-PAGE (Figure 1D and 1E). In synchronized erythrocytic stages, PyROM1-HA protein expression is lowest in ring stages (3 hours post invasion) and increases during the course of development with a peak in schizont stages (Figure 1D). In sporozoite stages, PyROM1-HA is readily expressed in salivary gland sporozoites with very minimal expression in midgut sporozoites. The expression pattern of PyROM1 at the mRNA and protein level implies a function at multiple stages of the parasite life cycle with a particular importance for the zoite stages that mediate host cell invasion.

### pyROM1 co-localization with secretory markers

**Figure 2:**
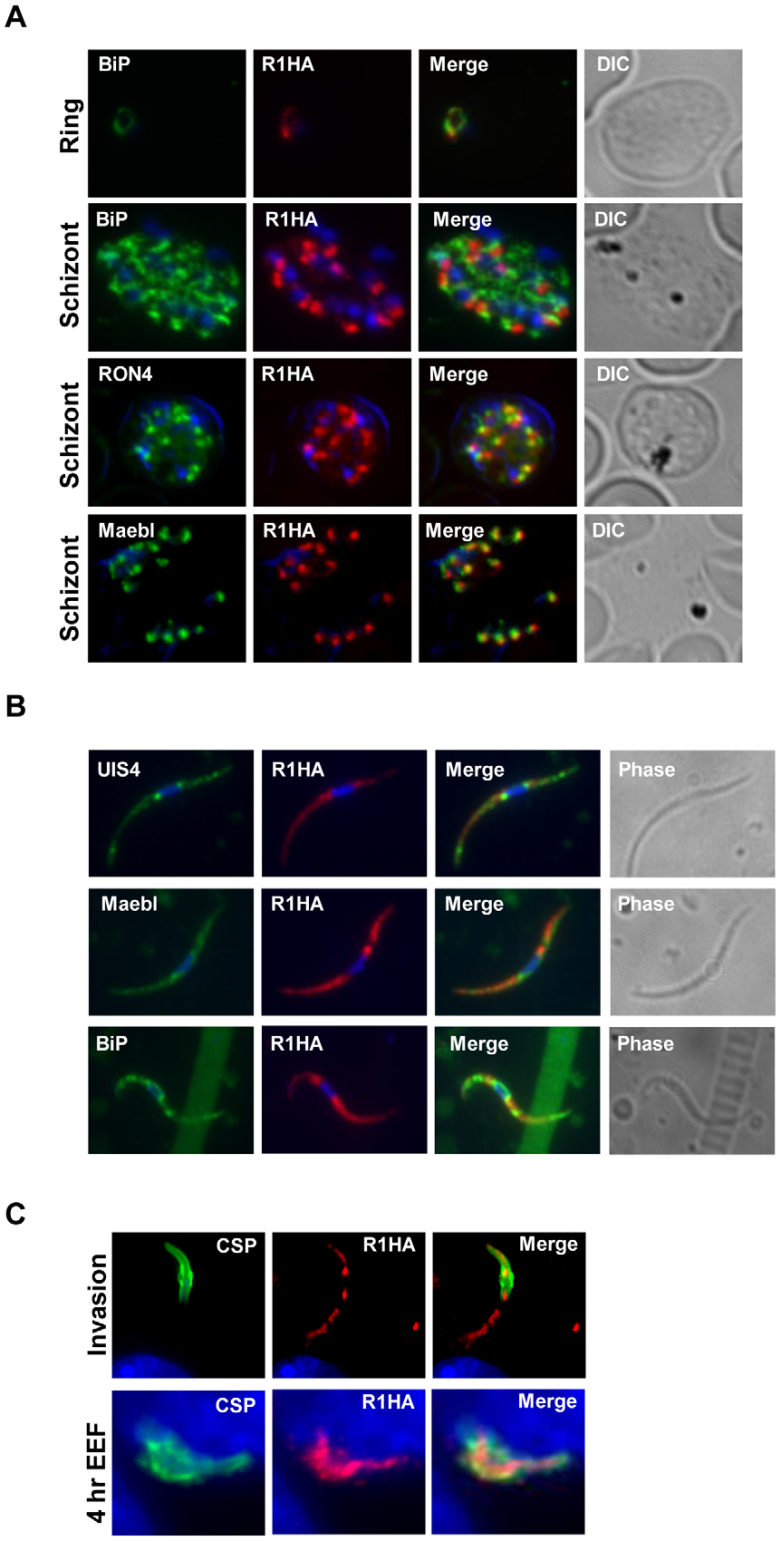
PyROM1 localizes with microneme markers and is not present at the parasite surface during sporozoite invasion. (A) Immunofluorescence assay of blood stages shows partial co-localization of PyROM1-HA (R1HA) with the microneme protein pyMAEBL and the rhoptry neck marker RON4 in schizont merozoites. PyROM1-HA also partially overlaps the ER marker BiP in schizonts. (B) Immunofluorescence assay of pyROM1-HA (R1HA) salivary gland sporozoites reveals that pyROM1 partially co-localizes with the microneme marker PyMAEBL and with the eTRAMP protein PyUIS4 within diffuse intracellular granules throughout the length of the parasite body. Restricted localization that excludes the area surrounding the nucleus is observed between PyROM1-HA and the ER marker BiP. (C) Localization of pyROM1-HA during invasion into Hepa 1–6 cells. The extracellular portion of parasite stains with CSP. Intracellular localization of pyROM1-HA in a developing EEF at 4 hours post invasion into Hepa 1–6 cells has overlap with CSP.

PyROM1-HA co-localizes with the microneme adhesin, pyMAEBL and with the rhoptry neck protein RON4 at the apical end of merozoites within mature schizonts by immunofluorescence (Figure 2A). Although localization overlapped more consistently with pyMAEBL than RON4, pyROM1 shows partial co-localization with both markers. Some localization overlap is observed between PyROM1-HA and the endoplasmic reticulum (ER) marker BiP (Figure 2A). PyROM1-HA is found in a speckled intracellular pattern in salivary gland sporozoites (day 14) that partially overlaps with localization of PyMAEBL and PyUIS4 (Figure 2B). Some co-localization is observed with BiP in salivary gland sporozoites (Figure 2B). During sporozoite invasion into Hepa 1–6 cells, PyROM1 remains prominent with a speckled pattern diffusely dispersed throughout the sporozoite (Figure 2C). Expression of pyROM1-HA is observed in developing EEFs at four hours post invasion with intracellular localization throughout the length of the parasite that is distinct from the localization of the sporozoite surface antigen, circumsporozoite protein (CSP) (Figure 2C).

### Genetic targeting of the gene encoding ROM1 in *P. yoelii*

**Figure 3:**
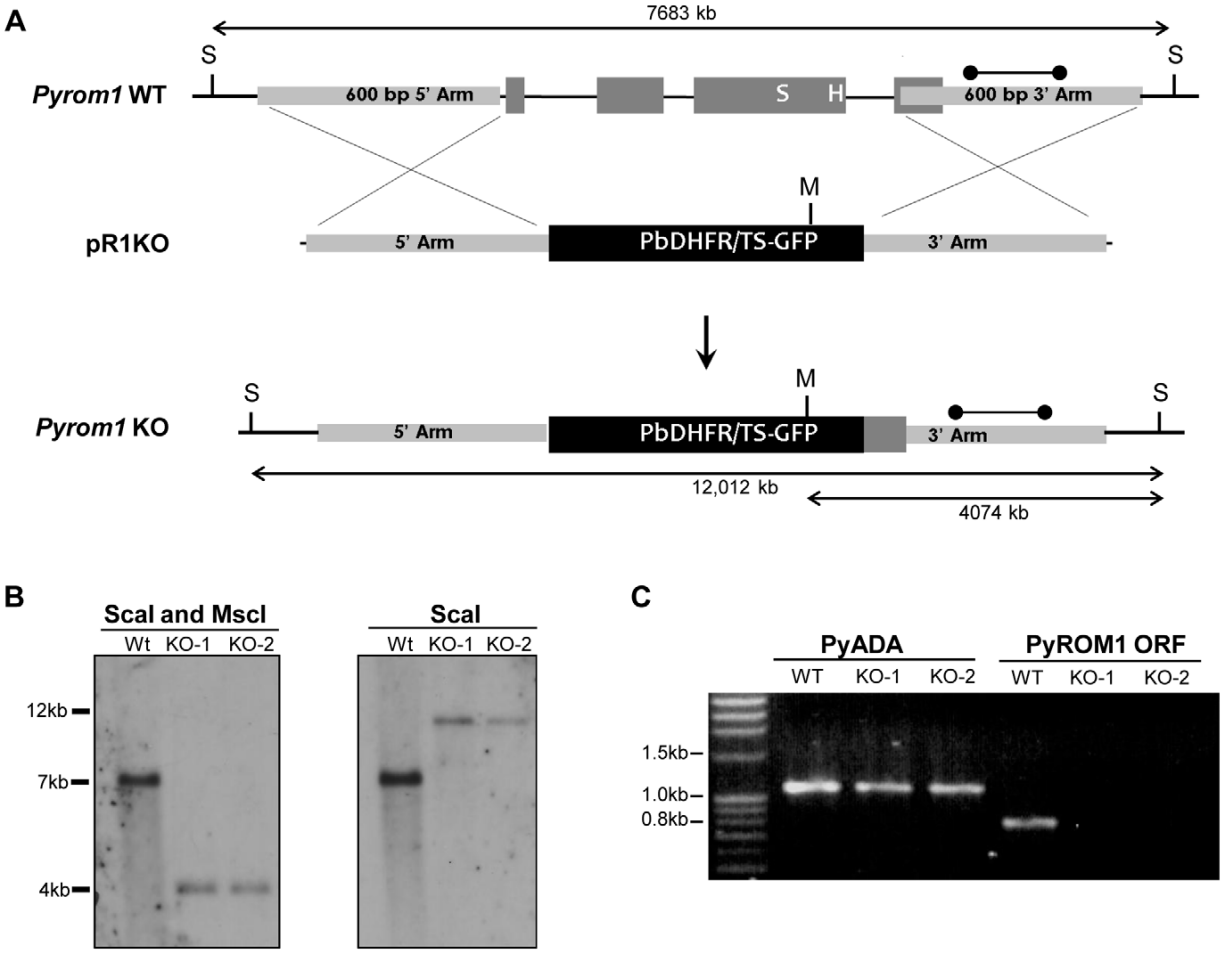
Generation of *pyrom1(-)* parasites by allelic exchange. (A) Schematic of targeting strategy using the targeting vector pR1KO which has the pbDHFR/TS-GFP selection cassette flanked by a 5′homologous arm (600 bp) and a 3′ homologous arm (600 bp) corresponding to the 5′ and 3′ regions of the *pyrom1* gene, respectively. After double cross over homologous recombination the endogenous *pyrom1* gene is replaced by the selection cassette resulting in parasites deficient for *pyrom1* (R1KO). (B) Proper gene replacement was verified by Southern Blot analysis in two knockout clones (KO1, KO2). Strategy for enzyme restriction digestion with either ScaI (S) or a combination of ScaI and MscI (M) using a probe (dumbbell bar) specific for the 3′ homologous arm is depicted in (A). A ScaI fragment that corresponds to the wildtype (Wt) genomic locus migrates at ∼7000 bp in both blots. Digestion of the recombinant locus (R1KO) with either ScaI/MscI or ScaI reveals expected bands migrating at the expected sizes of ∼4000 bp and ∼12,000 bp, respectively. (C) Lack of *pyrom1* gene expression in R1KO clones from mixed blood stages was verified by RT-PCR. Analysis of pyADA expression, an unrelated gene, was used as a positive control. Expression of *pyrom1* was analyzed using primers, R1-F and R1-R, which amplify the Open Reading Frame (ORF) of *pyrom1*. The expected *pyrom1* ORF band (expected size ∼800 bp) is readily amplified from wildtype (WT) cDNA, but is not amplified from cDNA of the two *pyrom1(-)* clones (KO-1 and KO-2).

To investigate the importance of *pyrom1* in the parasite life cycle, we employed a reverse genetics approach to generate loss-of-function deletion mutants. Initially we used a single crossover homologous recombination step to disrupt the endogenous *pyrom1* locus. Successful disruption of the *pyrom1* gene was confirmed by Southern blot and RT-PCR (Figure S3). Disrupted parasites (R1INT) were used to carry out initial phenotypic screens. Because single crossover disruptants can revert to wildtype [46]–[47] we also created a deletion mutant that would allow us to cycle the parasites between the mammalian host and mosquito vector without the risk of reversion. We used a gene replacement vector to exchange the endogenous *pyrom1* gene for a pbDHFR/TS-GFP cassette by double crossover homologous recombination [48]. The targeting vector contains the pbDHFR-TS/GFP selection cassette, which confers pyrimethamine resistance, flanked by DNA fragments from the upstream (5′ARM) and downstream (3′ARM) regions of the predicted open reading frame of *pyrom1*. We successfully integrated the replacement cassette, deleting exons 1–3 of the *pyrom1* gene that include the region encoding the functional catalytic serine motif and the conserved histidine (Figure 3A). Recombinant parasites were detected by diagnostic PCR of genomic DNA from wildtype and transfected parasites. Cloning of pyrimethamine-resistant parasites yielded two knock out clones (R1KO-1 and R1KO-2) and a wildtype clone (Wt Ctrl). Southern blot analysis of genomic DNA from mixed blood stages confirmed successful cloning of *pyrom1* deletion mutants (R1KO) (Figure 3B). RT-PCR analysis of mutant clones revealed no detectable *pyrom1* transcript expression, whereas *pyrom1* expression was detected in the wildtype control clone (Figure 3C). The successful generation of *P. yoelii* 17XNL parasites deficient in *pyrom1* demonstrates that this gene is not essential for proliferation of the intra-erythrocytic stages.

### *Pyrom1(-)* parasites are attenuated in the mammalian host

**Figure 4:**
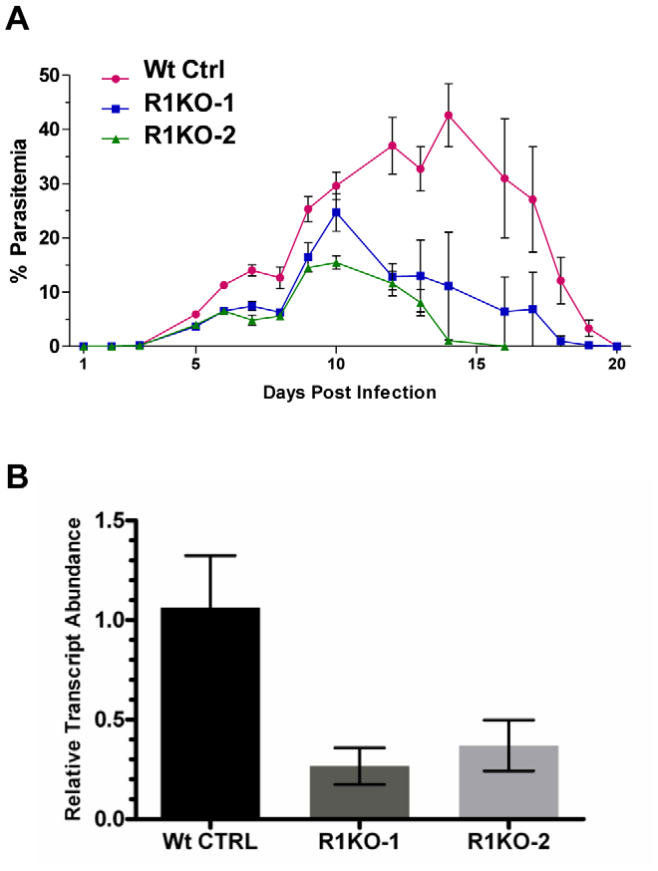
Phenotypic analysis of *pyrom1(-)* mutant parasite in the mammalian host. (A) Female Balb/c mice, 6 weeks of age (N = 5) were infected with mixed blood stage parasites from either *P. yoelii* 17XNL wildtype control (Wt Ctrl) or *pyrom1(-)* clones R1KO -1 and R1KO-2 by tail intravenous injection with 1×10^4^ iRBC. Percent parasitemia (infected Red Blood Cells/Total Red Blood Cells ± Std) was calculated on a daily basis from Giemsa stained smears counting at least 1000 total Red Blood Cells per smear. Values presented are the mean parasitemia/day from each infected group with the error as the standard deviation. Parasitemia was measured until the mice had cleared the infection. (B) Salivary gland sporozoites (SPZ), 10^4^, were injected intravenously into 6 week Balb/c female mice (N = 5). At 36 hours post infection, whole livers were taken RNA was extracted for quantitative RT-PCR analysis. Liver burdens were determined as the levels of *Plasmodium* 18S rRNA transcript expression normalized to mouse GAPDH expression levels. Liver burden, defined as the level of parasites in liver based on 18S expression levels, was decreased in the two *pyrom1(-)* parasite (R1KO-1 and R1KO-2) infected mice relative to mice infected with wildtype (Wt Ctrl) parasites. Fold change was calculated using the ddCt (2^−ddCt^) method and relative transcript levels are shown.

To test whether PyROM1 serves an important function during asexual growth within red blood cells, we performed *in vivo* infectivity assays. Six-week old female BALB/c mice (n = 5) were injected intravenously with 1×10^4^ infected red blood cells (iRBCs) and parasitemia was monitored every 24 hours by counting Giemsa-stained blood smears. Since the *pyrom1(-)* parasites were generated in *P. yoellii* 17XNL, a non-lethal strain, the parasitemia in mice reached a peak and parasites were then cleared by the mouse immune system. We monitored parasitemia until infected erythrocytes were undetectable. Growth curves revealed a mild attenuation of *pyrom1(-)* (R1KO) with a decrease in peak parasitemia (25% versus 43%) and a decrease in the duration of infection compared to the wildtype control (Wt Ctrl) (Figure 4A).

Infection of BALB/c mice (n = 5) with salivary gland sporozoites by natural mosquito bite or by intravenous injection (inoculum of 20–2000) revealed that mutant parasites could establish a liver infection leading to blood stage infection with a pre-patent period of three days (Table S1). Therefore, *pyrom1(-)* parasites are able to progress through the entire lifecycle. Due to the highly infectious nature of *P. yoelii* sporozoites *in vivo*, it is difficult to quantify liver development by relying solely on pre-patency period [49]. Therefore, we analyzed the development of *pyrom1(-)* sporozoites *in vivo* using quantitative RT-PCR, a more sensitive assay to quantify hepatic development [50]. BALB/c mice were injected intravenously with equal numbers (1×10^4^) of salivary gland sporozoites from wildtype control or *pyrom1(-)* parasites. Livers were harvested at 36 hours and RT-qPCR analysis was carried out. Parasite burden in mice infected with *pyrom1(-)* parasites was decreased by at least 60% compared to mice infected with wildtype parasites (Figure 4B). Similar results were seen with livers harvested at 24 hours and 42 hours from mice infected with 10^4^ salivary gland sporozoites (data not shown). This difference in liver burden suggests that pyROM1 is important for efficient sporozoite infection in the liver.

### *Pyrom1* is not essential for transmission through the mosquito vector

Since *pyrom1* is robustly expressed in sporozoite stages, we investigated the effect of pyROM1 depletion in the mosquito phase of the parasite life cycle. Analysis of blood smears showed no difference in the capacity to produce gametocytes between wildtype and R1KO parasites (Table 1). No significant difference was seen in the number of oocysts (day 8 post infection) per mosquito midgut or in the prevalence of infected mosquitoes between wildtype and R1KO parasites (Table 1). Furthermore, there was no difference in the number of salivary gland associated sporozoites (day 14 post infection) in R1KO mutant parasites compared to wildtype in three independent experiments, testing two R1KO clones (Table 1). Thus, deletion of *pyrom1* does not affect development of oocysts, sporozoite release into the hemolymph, or invasion of salivary glands.

**Table 1:** *PyRom1(-)* parasites progress normally through the mosquito vector.

Exp.	Mouse Line	% Gct^a^	Mean Oocyst/msq^b^	N^c^	Prevalence^d^	p-value vs. Ctrl^e^	Sporozoites/Msq^f^
**1**	WT Ctrl	0.54±0.22	69±18.10	18	83%	-	23,470±3965
	R1KO-1	0.37±0.14	107.6±22.00	14	93%	P>0.05	21,437±1738
	R1KO-2	0.46±0.00	51.63±11.32	19	84%	P>0.05	21,772±561
**2**	WT Ctrl	0.62±0.19	34.5±11.19	18	100%	-	28,759±3960
	R1KO-1	0.74±0.45	35.17±10.33	18	94%	P>0.05	24,780±184
	R1KO-2	0.44±0.12	40.94±10.33	17	88%	P>0.05	23,255±648
**3**	WT Ctrl	0.62±0.22	70.5±13.14	24	100%	-	20765.5±477
	R1KO-1	0.35±0.10	64.44±16.81	16	93%	P>0.05	19534±854
	R1KO-2	0.36±0.15	43.73±9.647	22	91%	P>0.05	18947±1654

a)Percent gametocytemia calculated per mouse used during mosquito feeding. Shown is the mean with standard deviation.

b)Mosquito midguts dissected at day 8 post feeding. Mean number oocysts per midgut reported with Standard Error Mean.

c)Number of midguts dissected and counted per parasite line for each experiment.

d)Prevalence is calculated as the percentage of infected midguts over total midguts analyzed.

e)Statistical analysis using non parametric 2-way ANOVA/Tukey's post test with Prism Graph Pad.

f)Salivary glands dissected at day 14 post feeding. Mean number of sporozoites/mosquito is reported ± standard deviation.

### *Pyrom1(-)* salivary gland sporozoites glide and invade host cells normally

**Figure 5:**
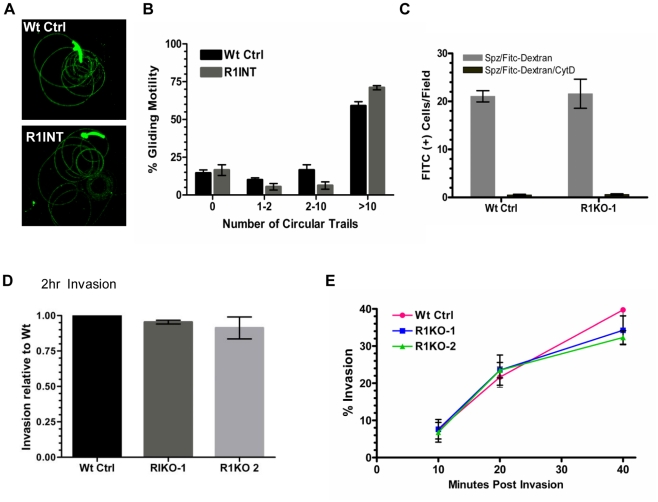
*Pyrom1(-)* salivary gland sporozoites have no defect in gliding, traversal, or host cell invasion. (A) Salivary gland sporozoites from wildtype or the *pyrom1* disrupted parasite line (R1INT) were allowed to glide on glass and gliding trails of deposited CSP were stained were visualized under fluorescent microscope (B) Quantification of sporozoite trails was carried out in triplicate and at least 25 fields were counted per well. The number of circular trails were quantified per sporozoite and shown is the mean percentage (± SD) of sporozoites that were associated with 0, 1–2, 2–10, or >10 circular trails. (C) Salivary gland sporozoites were loaded on top of a monolayer of Hepa 1–6 cells in the presence of 1 mg/ml FITC-dextran and allowed to traverse cells. As an internal negative control, sporozoites were treated with the microfilament inhibitor Cytochalasin D (CytD) prior to loading on top of Hepa 1–6 cells. The numbers of FITC-positive cells/field were counted under the microscope. The mean number of fluorescent cells/field (± SD) is shown for a representative. (D) Salivary gland sporozoites were loaded on Hepa1–6 monolayers and allowed to invade for two hours. A double staining assay was carried out to distinguish intracellular versus extracellular parasites. The % invasion was calculated as the number of (Green - Red parasites)/(Green parasites). Values presented are the mean number counts relative to the wildtype control (set to 1) ± standard deviation. (E) Kinetics of invasion was analyzed at three time points (10 min, 20 min, 40 min) during early invasion using the double staining assay described in (D). Y-axis values represent the % invasion of partially invaded or fully invaded parasites ± standard deviation.

A decrease in liver stage infection *in vivo* can be attributed to a defect in sporozoite motility or cell traversal ability [51]–[55]. Potential rhomboid substrates such as the microneme adhesin, TRAP, bridge the function of gliding motility and host cell invasion [56]–[57]. To begin an in-depth analysis of the function of *pyrom1* in salivary gland sporozoites, we tested gliding motility and cell traversal of *pyrom1(-)* parasites. Gliding trails formed by the deposition of circumsporozoite protein (CSP) on glass slides by sporozoites were readily detectable in the *pyrom1* disrupted (R1INT) parasites (Figure 5A) and the quantity and quality of trails produced by wildtype and R1INT sporozoites were similar (Figure 5B).

A unique feature of salivary gland sporozoites is their ability to use the motility machinery to traverse cells without productively invading a host cell and forming a PV [58]. When sporozoites traverse cells they glide in and out of the cell puncturing the plasma membrane and causing host cell injury. Host cell traversal can be detected by quantifying host cells that take up non-permeable high molecular weight FITC-dextran, in the presence of migrating sporozoites [59]. Using this assay, there was no difference in cell traversal activity between wildtype or R1KO sporozoites (Figure 5C).

To test the importance of pyROM1 during host cell invasion, we performed a double labeling invasion assay that distinguishes parasites that are intracellular versus parasites that are extracellular [60]–[61]. The number of intracellular sporozoites between wildtype and *pyrom1(-)* parasites did not differ at two hours post invasion (Figure 5D). Invasion rates at 10, 20, and 40 minutes after loading onto Hepa1–6 monolayers did not differ significantly between Wt Ctrl and R1KO, illustrating no significant difference in the kinetics of invasion between wildtype and R1KO sporozoites into Hepa1–6 cells (Figure 5E). Together, these results indicate that pyROM1 is not important for efficient host cell entry or for establishment of a successful infection during the early stages of sporozoites in host cells.

### *Pyrom1(-)* parasite development in hepatocytes decreases in the first 24 hours

**Figure 6:**
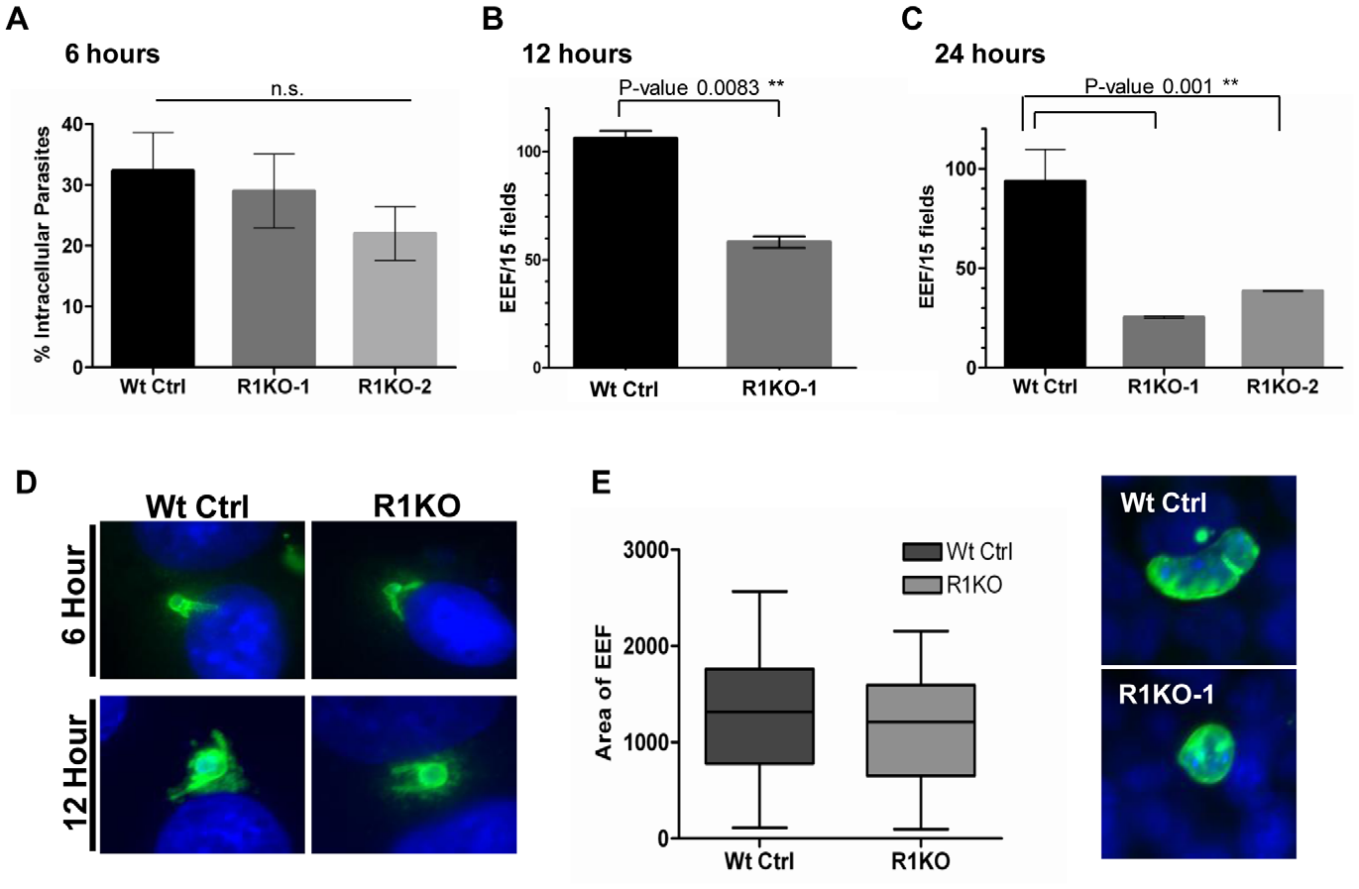
Development of *pyrom1(-)* EEF is significantly reduced within the first 24 hours of hepatic development. Development of sporozoites within Hepa 1–6 cells was analyzed at 6 hours, 12 hours, and 24 hours post invasion. (A) A double staining assay was carried out to determine the number of intracellular parasites undergoing development inside Hepa1–6 cells at 6 hours post infection The % intracellular parasites was determined by counting at least 30 fields per well. Values shown are the mean % intracellular parasites from duplicate counts ± standard deviation. The values were not statistically significant (P-value 0.17) using unpaired t test analysis. Parasite development at 12 hours (B) and 24 hours (C) post infection was assayed by staining with α-pyCSP and developing parasites per field were counted (at least 30 fields were counted per well). The numbers represented are the mean values of developing parasites/15 fields ± standard deviation. At 12 hours development there is a 45% reduction in *pyrom1(-)* parasite development (P-value 0.008 using one-way ANOVA analysis) and at 24 hours there is at least a 60% reduction in pyrom1(-) parasite development (P-value 0.001 using one-way ANOVA analysis) D) Images of developing liver stage parasites at 6 hours and 12 hours stained with CSP show that despite reduced numbers, R1KO parasites are capable of undergoing the initial differentiation, transformation, and sphericalization during early hepatic development. (E) Development of *pyrom1(-)* parasites that survive the initial 24 hours go on to develop normally. Immunofluorescence of exo-erythrocytic forms (EEF) at 40 hours post invasion of Hepa1–6 cells was performed using anti-pyCSP to visualize developing parasites. Pictures of at least 50 EEF/well (duplicate) were taken. Area of EEF was measured using Image J software. Area was determined as an arbitrary value from images taken with the same magnification (40×). Bar graphs represent the mean area ± SEM.

Analysis and quantification of sporozoite development in Hepa 1–6 cells at 6, 12, and 24 hours post invasion was carried out using fluorescence microscopy. Differential red-green staining of pyCSP was carried out to distinguish intracellular versus extracellular parasites at 6 hours post invasion. By 6 hours, exoerythrocytic forms (EEF) have begun to round up and change morphology as a prelude to development in hepatocytes. The percent of R1KO developing sporozoites in Hepa 1–6 cells was decreased at 6 hours compared to wildtype sporozoites (Figure 6A) but this difference was not statistically significant (P-value 0.17). At 12 hours, the number of developing foci of R1KO exoerythrocytic forms (EEFs) had significantly decreased by 45% compared to the number of wildtype EEF foci (P-value 0.008; Figure 6B). By 24 hours post infection, R1KO EEF development was decreased by more than 60% compared to wildtype EEF development (P-value 0.001; Figure 6C). This time course analysis of EEF development suggests that pyROM1 enhances the survival of developing sporozoites within hepatocytes during the critical stages of early intra-hepatocytic development.

Although the number of developing EEFs decreased with each time point, the R1KO EEFs that were observed still exhibited morphological characteristics of parasites that had progressed appropriately during development. For example, these EEFs exhibited rounding up of the elongate sporozoite, increase in cell size, and juxta-nuclear position within host cell (Figure 6D). To determine the fate of the fraction of parasites that survive past the initial 24 hours post infection, we allowed parasites to develop in Hepa1–6 for 40 hours. By this time, the EEF has grown in size and has undergone several rounds of multiplication, becoming a liver schizont (i.e. mature EEF). Liver schizonts were analyzed microscopically by staining with CSP to determine if pyROM1 plays a role during growth and later development stages of EEF maturation. Quantification of EEF area reveals that R1KO parasites that survived the initial stages of development are able to proceed with normal growth and cell division (Figure 6E).

### The parasitophorous vacuole protein, UIS4, is a rhomboid substrate and its association with the PV is decreased in *Pyrom1(-)* parasites

**Figure 7:**
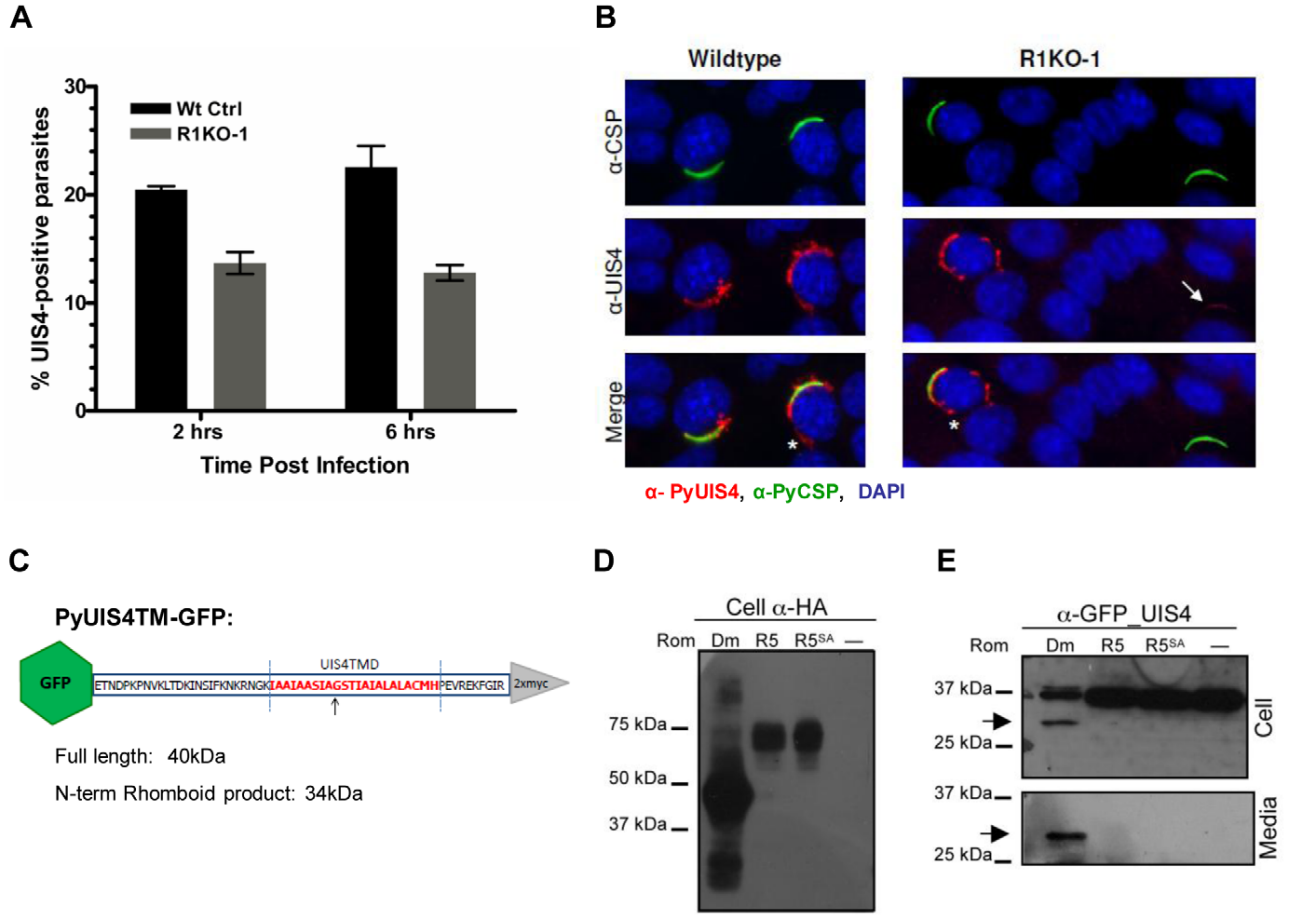
The parasitophorous vacuole protein PyUIS4 is a rhomboid substrate and its association with the PVM is decreased in a fraction of *Pyrom1*(-) parasites. (A) Immunofluorescence assay was performed on methanol fixed and permeabilized parasites post hepatocyte invasion at 2 hrs and 6 hrs development. The parasitophorous vacuole membrane (PVM) was detected by staining with α-pyUIS4. Parasites (both extracellular and intracellular) were stained with pyCSP. The percent UIS4-CSP double stained parasites was determined by counting parasites staining with both UIS4 (PVM staining; see B) and CSP and dividing this number by the number of parasites staining with CSP (total parasites including extracellular parasites). Numbers are the average of triplicates ± SEM (at least 300 parasites counted) and were statistically significant using the unpaired t test analysis. (B) Images from experiment in (A) of sporozoites at 2 hours post Hepa1–6 infection showing localization of PyUIS4 (red) within PVM and the associated tubulovesicular network (asterisk) characteristic of intracellular developing parasites versus the speckled UIS4 localization within the sporozoites (white arrow). In the quantification of (A), only parasites with UIS4 PVM staining were counted positive for UIS4. Anti-CSP antibody (green) was used to visualize al parasites (intracellular and extracellular). The nucleus was stained with DAPI (blue). (C) Schematic of PyROM4TMGFP construct displaying the truncated sequence of PyROM4 used to clone into a vector containing an IgK leader sequence, an N-terminal GFP tag (green hexagon), and a C-terminal 2xMyc tag (gray triangle). Dashed blue parallel lines represent the boundaries of the TMD (amino acid sequence in red font color) as predicted by HMMTOP and TMHMM analysis tools (Expasy.org). Arrow depicts the predicted rhomboid cleavage site between an Alanine and a Glycine. Predicted full length size for PyROM4TMGFP is 40 kDa, and the predicted size for the N-terminal rhomboid cleaved product is a 34 kDa. (D and E) Western blot analysis of transiently transfected COS7 cells used to detect proteolytic activity of rhomboid against PyUIS4TMGFP. (D) Western blot of cell lysates probed with anti-HA antibody (Cell α-HA) show proper expression of DmRho-1 (Dm), TgROM5 (TgR5), and TgROM5^SA^ (TgR5^SA^) in the first three lanes and no detection of protein in the “no rhomboid” (–) negative control in the fourth lane. (E) Processing of PyUIS4TMGFP (anti-GFP_UIS4) was analyzed by western blot of cell lysates (upper panel, Cell) and concentrated media fraction (lower panel, Media) by probing with anti-GFP antibody. Full length PyUIS4GFP is observed in the cell fraction at the expect size of ∼40 kDa. A lower molecular weight product (∼34 kDa) is detected in both cell and media fractions (arrow) only in the first lane where DmRho-1 (Dm) is co-expressed. No cleaved product is seen when PyUIS4TMGFP is co-expressed with TgROM5 (TgR5), TgROM5^SA^ (TgR5^SA^), and the “no rhomboid” negative control (–).

Proper establishment and modification of the PV in newly invaded parasites is a prerequisite for intracellular survival and growth. The early transcribed membrane proteins (eTRAMPs) are highly charged type I integral membrane proteins that localize to secretory organelles and associate with the PVM shortly after parasite invasion [15], [62]. The eTRAMP UIS4 (Upregulated in Sporozoites 4) is specifically upregulated in salivary gland sporozoites and is essential for parasite development in host hepatocytes [24]. In salivary gland sporozoites, UIS4 co-localizes with TRAP to secretory organelles [15] and is secreted at some point post invasion (within 2 hours) to become incorporated into the PVM [24], [63]. To assess whether the defect in *pyrom1(-)* parasite development in host hepatocytes was due to a defect in PV formation, we tested for the presence of PyUIS4. Sporozoites within hepatocytes were labeled with CSP mAb and UIS4 antisera at 2 hours and 6 hours post infection and quantified microscopically. The total number of parasites (intracellular and extracellular) was determined by CSP staining. The percent of developing parasites surrounded by a UIS4-positive PVM was calculated as the number of parasites that showed double staining with UIS4 and CSP. R1KO parasites had a 33% and 43% decrease compared to wildtype parasites of UIS4 positive staining at 2 hours and 6 hours, respectively (P-value 0.010) (Figure 7A). *Pyrom1(-)* parasites negative for the circumferential staining typical of UIS4 localization to the PVM, often times had a punctuate localization of UIS4 within the sporozoite body (Figure 7B).

The eTRAMP proteins possess a conserved TMD with sequence similarity to known rhomboid substrates [32]. To test if the TMD of UIS4 is a potential rhomboid substrate we carried out a previously established heterologous cleavage assay where rhomboid and substrate are transiently co-expressed in COS7 cells and analyzed via Western blot [39]. A truncated version of PyUIS4 encompassing the full TMD with an N-terminal GFP tag and an IgK leader sequence (UIS4TMGFP, Figure 7C,Table S3) was co-expressed with HA-tagged rhomboid protease. Proper expression of rhomboid proteases was observed in Western blots probed with anti-HA antibody (Figure 7D). A single band running at ∼40 kDa corresponding to full length UIS4TMGFP was observed in Western blots of cell lysates probed with anti-GFP antibody (Figure 7D). A lower molecular weight band (∼34 kDa) corresponding to the expected size of a rhomboid cleaved product was observed in cell lysates and conditioned media only when UIS4TMGFP was co-expressed with the *D. melanogaster* rhomboid protease, DmRho-1 (Figure 7D). No cleavage activity was readily observed when UIS4TMGFP was co-expressed with either TgROM5 or its catalytic mutant (TgROM5^SA^) (Figure 7D). Therefore, it can be concluded that PyROM4, and potentially other eTRAMPs, can serve as substrates to rhomboid proteases.

Our analysis of UIS4 in *Pyrom1(-)* parasites show that there is an inability to properly secrete UIS4 into the PVM. Furthermore, a rhomboid protease may be involved in the proper maturation, processing and targeting of PyUIS4 to the PVM since the TMD of UIS4 serves as a rhomboid substrate.

### Electron microscopic analysis of *Pyrom1(-)* parasites reveals a difference in PV formation

**Figure 8:**
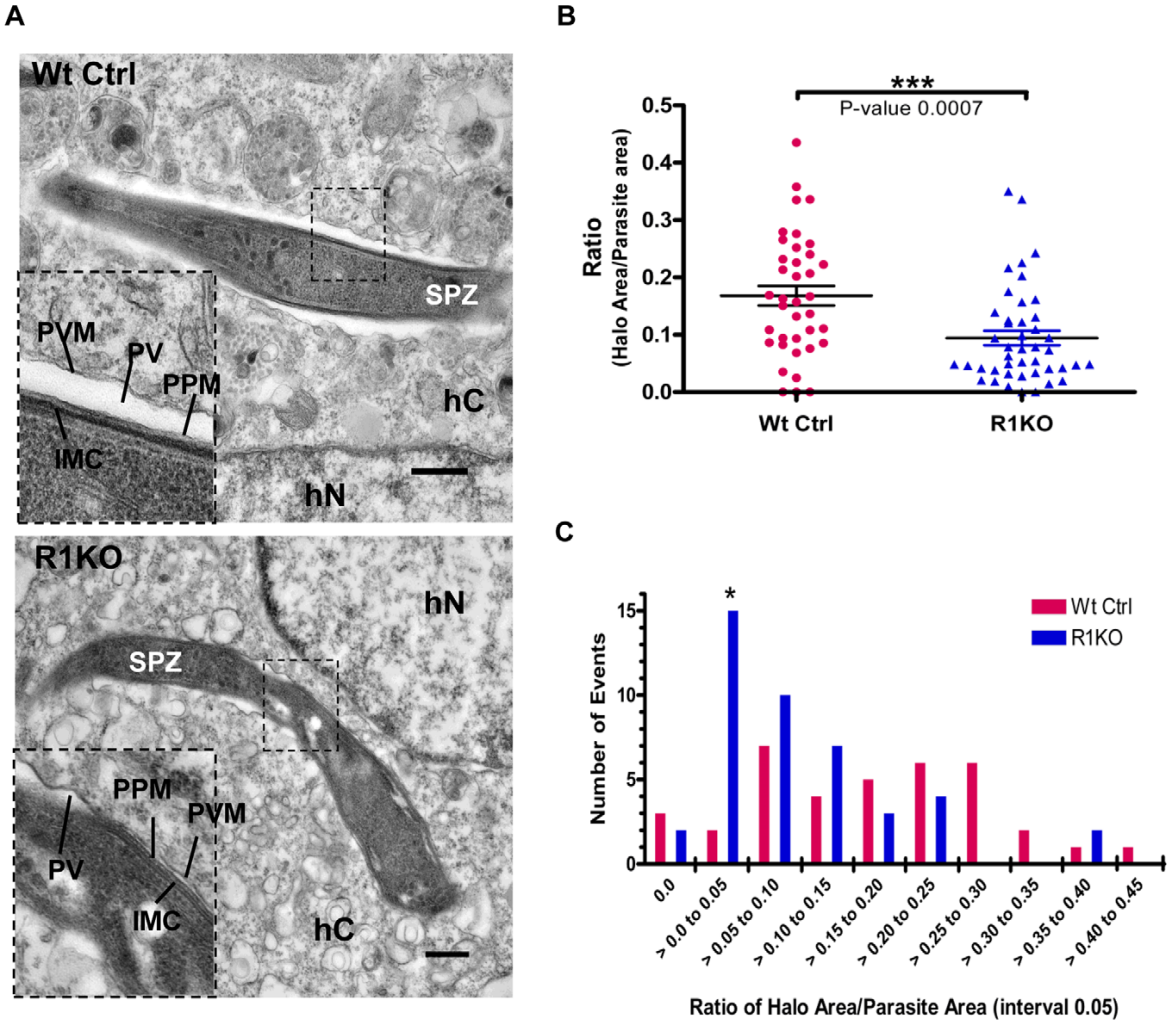
Ultrastructural analysis of *pyrom1(-)* parasites reveals an abnormal parasitophorous vacuole. (A) Representative electron micrograph images of intracellular parasites developing within a Hepa 1–6 host cell at 4 hours post invasion. Inset shows a close up of the boundary between the parasite and the host cell cytoplasm. Notice the expanded PV space appearing like a halo (*) in the wildtype parasite and a tight fitting PVM with reduced PV space in the R1KO parasite. Scale bars are equal to 0.5 µm. Abbreviations: SPZ-sporozoite, hC- Host cell cytoplasm, hN- Host cell nucleus, PVM- Parasitophorous vacuole membrane, PV-Parasitophorous vacuole space, PPM- Parasite Plasma Membrane, IMC- Inner Membrane Complex. (B)) Quantification of PV space area (halo area) and parasite area was carried out using ImageJ software. The ratio of halo area/parasite area ± SEM for Wildtype parasites (Wt Ctrl) was 0.168±0.017 (N = 38) and for R1KO parasites was 0.094±0.013 (N = 44). Statistics was carried out using One-way ANOVA analysis. (C) The graph shows the distribution of the parasite population as a function of the ratio halo area/parasite area between wildtype (Wt Ctrl) and *pyrom1(-)* (R1KO). A shift in the R1KO population towards a smaller ratio (>0.00 to 0.05) is observed and is denoted by an asterisk (*).

To investigate whether *pyrom1(-)* parasites have a defect in PVM formation, we carried out ultrastructural analysis of R1KO parasites during early intra-hepatocytic development. Intracellular parasites at 4 hours post infection of Hepa1–6 cells were analyzed by electron microscopy. R1KO parasites (19 of 42) exhibited a strikingly reduced intravacuolar space with the PVM in close apposition to the plasma membrane of the parasite. In wildtype vacuoles this parasitophorous vacuole space has expanded and appears as a white “halo” surrounding the developing parasite inside a PVM and only one of 38 vacuoles had a closely apposed PVM Figure 8A, Figure S4, Table S2). To quantify this phenotype we measured the area of the PV space (halo) as the difference between the area of the total PV space and the parasite area. This value was then normalized to the area of the parasite to give a ratio that represents the percentage of the PV space area relative to the parasite and is a representation of the extent of PV space expansion. Wildtype parasites had a significantly (P-value 0.0007) larger ratio value (0.17±0.02) compared to R1KO parasites (0.09±0.01) (Figure 8B). To quantitatively show the existence of two populations, we plotted the distribution of parasites as a function of PV space area/parasite area ratio. The distribution plot clearly shows the existence of two parasite populations within the R1KO group (Figure 8C). We hypothesize that the R1KO parasites with a normal PV space go on to develop past the initial 24 hours of development and the R1KO parasites with a reduced PV space abort development within the initial 24 hours. A few parasites in direct contact with the cytoplasm or nucleoplasm were observed in both wildtype (4/38) and R1KO (2/42) parasites, which we assume be sporozoites in traversal mode [64]. This ultrastructural analysis indicates that while R1KO parasites are capable of productively invading host cells with the formation of a PVM, a fraction of them have a defect in the subsequent PV expansion and modification in early development.

## Discussion

Successful transmission of malaria into the mammalian host is dependent on the ability of sporozoites to invade and establish a proper PV within the host hepatocyte. To date, only a handful of genes have been identified that play a role during invasion or early development of the sporozoite within hepatocytes. In this study we have characterized the rhomboid protease, ROM1, throughout the lifecycle of the malaria rodent model, *Plasmodium yoelii*.

Quantitative expression analysis of *pyrom1* shows it is expressed at various invasive stages of the malaria life cycle. Expression of *pyrom1* follows a pattern similar to genes involved in merozoite invasion with maximal erythrocytic stage expression in schizonts. Relative to schizont stages, expression of *pyrom1* is increased by at least 10-fold during the sporozoite stages with *pyrom1* transcript levels upregulated by 2-fold from midgut sporozoites to salivary gland sporozoites. Midgut sporozoites are substantially less infectious in the mammalian host compared to salivary gland sporozoites [14], [65]. This gain of infectivity has been ascribed in part to transcriptional changes that occur when sporozoites invade mosquito salivary glands [14]–[15]. In addition, we observe that, although transcript expression of *pyrom1* is elevated in midgut sporozoites, protein expression is barely detectable by Western blot, suggesting regulation of *pyrom1* gene expression at the level of transcript translation in midgut sporozoites. The upregulated expression of ROM1 mRNA and protein in salivary gland sporozoites is consistent with a role during infectivity and transmission into the vertebrate host. Supporting our observations, a microarray study looking at gene expression changes triggered by different host environments (i.e. mosquito host to mammalian host) reported that *pfrom1* is upregulated 4-fold when salivary gland sporozoites are shifted to 37°C in the presence of hepatocytes [66].

We have localized PyROM1 at various stages during the malaria life cycle. In schizonts, PyROM1-HA is localized to the apical end. In salivary gland sporozoites PyROM1-HA has a diffuse granular staining pattern similar to microneme proteins such as PyMAEBL. Microneme localization of PyROM1 agrees with previous studies of ROM1 localization in both *Toxoplasma gondii* and *Plasmodium spp* [67]–[68], [41]–[43], [29], but in our study this localization was only partial. Localization of pyROM1-HA in sporozoites during invasion revealed that it remains intracellular and does not seem to be secreted onto to the surface during invasion. There is no consensus on the localization of ROM1 during apicomplexan invasion since some studies report surface expression and posterior translocation [40], [43] and others report internal localization during invasion [29], [41]. Because of differences in the experimental conditions and expression constructs used for these localization studies, results are difficult to compare.

Using a gene deletion approach, we demonstrate that pyROM1 functions during intracellular growth, within hepatocytes and erythrocytes, to provide a fitness advantage. Quantitative analysis of parasite development within Hepa1–6 cells revealed that PyROM1 is not essential for sporozoite invasion into the host cells. Instead, survival of *pyrom1(-)* parasites decreases within the first 24 hours post invasion. It is during these first 24 hours of development that *Plasmodium* sporozoites undergo critical morphological changes such as PV modification, sphericalization of the elongate sporozoite, and increase in cytoplasmic size [18]–[19], [69]. PyROM1 may facilitate these initial vital steps during liver stages by allowing parasites to reach the critical threshold required to survive past the first 24 hours of differentiation. This *in vitro* development phenotype was confirmed *in vivo* when mice infected with *pyrom1(-)* sporozoites had decreased parasite liver burden at 36 hours post infection.

Our results agree with previous studies showing that lack of ROM1 expression causes a decrease in parasite survival [41]–[42]. In a previous study, disruption of *pbrom1* resulted in attenuation during erythrocytic stages, a decrease in oocyst formation, and a decrease in liver stage burden [42]. One major difference between the current study and the Srinivasan *et al* study is that we did not detect a decrease in the number of oocyst during development in the mosquito midgut. This difference in phenotypic observation can be attributed to the different methodologies used to analyze this stage. Oocyst development is biologically variable due to multiple factors such as host immunology (mosquito and mammalian), parasite variability, biological bottle necks, and other environmental conditions. Therefore, it is imperative that careful and repeated quantification and analysis of oocyst development be carried out in order to obtain statistically reproducible results. We have analyzed oocyst development in *pyrom1(-)* parasites thoroughly through multiple repeated experiments (at least three independent experiments) performed simultaneously with wildtype controls, using different batches of mosquitoes, and multiple parasite clones.

We have analyzed the development of *pyrom1(-)* parasites in hepatocytes thoroughly by using well-established *in vitro* development and invasion assays. Thus our studies have extended our understanding of ROM1 function and have pinpointed more precisely the role of ROM1 during intracellular development. Brossier et al, studied ROM1 in *Toxoplasma gondii* tachyzoites and found that TgROM1 knockdown parasites had a 50% reduction in the number of daughter tachyzoites per vacuole [41]. Based on measurement of EEF area, we did not detect a defect in *pyrom1(-)* parasite growth per vacuole, but an overall decrease in the number of developing parasites. Therefore, we predict that the intracellular parasites abort development in the stages preceding multiplication. This difference in phenotype between the TgROM1 knockdown parasites and our *pyrom1(-)* parasites may be explained by fundamental biological differences in the mode of replication between the two genera. *Toxoplasma gondii* tachyzoites divide by endodyogeny, a process where two daughter cells are formed within a mother cell, whereas *Plasmodium* parasites divide by schizogony a process where nuclear division leads to the formation of a multinuclear syncytium followed by the budding off of daughter merozoites at the periphery [70]. Thus, the events that occur post-invasion may have differing effects on the subsequent survival and development of the respective parasites [71].

The malaria parasite has four invasive stages: the ookinete, the midgut sporozoite, the salivary gland sporozoite, and the erythrocytic stage merozoite. Only two of these invasive zoites, the merozoite and the salivary gland sporozoite, form a PV, within which, growth and division of the parasite ensues. The phenotype displayed by *pyrom1(-)* parasites is only observed during the two stages where PV formation is a prerequisite for growth. The PVM provides a barrier that protects the parasite from host cell defenses such as lysosomal clearance or autophagy [72]–[73]. This barrier is also the portal for nutrient acquisition and communication with the external environment. The intravacuolar parasite must modify and rearrange the PV through the secretion of contents from rhoptries and dense granules, with molecules that are yet to be fully characterized. The exclusivity of the *pyrom1(-)* phenotype during intracellular development within the mammalian host suggests that ROM1 function is linked to proper PV formation and maturation either during or post invasion.

Ultrastructural analysis at four hours post invasion reveals that *pyrom1(-)* parasites are capable of forming a PVM during hepatic development. But, the PVM in the *pyrom1(-)* parasites is intimately associated to the plasma membrane of the mutant parasite. In comparison, after invasion, wildtype parasites have substantially expanded their PV space, which is visible as a white ‘halo’ surrounding the intracellular parasite. When parasites first enter host cells, they have a tight fitting vacuole. Shortly after invasion, the vacuolar space expands, representing a modification of the PV. This modification is accompanied by targeting of parasite proteins to the PVM as well as changes in the lipid composition of the specialized vacuolar membrane [12].

Notably, a fraction of *pyrom1(-)* parasites had a PV space similar to the wildtype parasites. We hypothesize that these are the parasites go on to develop normally into liver schizonts. These parasites may be able to progress due to a redundant function of other proteases such as ROM4 or ROM8, which are also expressed in sporozoites [44], [66]. Thus, *pyrom1 (-)* parasites display a partial penetrance phenotype where only a fraction (50–70%) of the clonal population display a mutant phenotype and the remainder fraction go on to develop normally. In addition to redundant function and partial complementation by another protease, this partial penetrance phenotype is explained by a stochastic mechanism where levels of processed substrate(s) must reach a threshold level to allow normal parasite development [74]–[76]. In systems biology, such a stochastic mechanism occurs when a gene encodes for a non-essential phenotypic capacitor that serves to buffer various development, environmental, and genetic stresses. Since PyROM1 encodes an enzyme, it is subject to random biomolecular interactions that promote phenotypic variation.

A previous study showed that intracellular parasites, deficient in two sporozoite specific proteins, p52 (p36p) and p36, were negative for UIS4 staining at the PVM [63]. Like *pyrom1(-)* parasites, mutant parasites in p36/p52 have normal gliding, traversal, and hepatocyte invasion. However, unlike *pyrom1(-)* parasites, which are capable of establishing a PVM, p52 mutant and p36/p52 double mutant parasites failed to develop inside the hepatocyte due to an inability to establish or maintain a PVM during early infection [26], [63], [77]–[79]. Interestingly, *pyrom1(-)* parasites have a 30% decrease in UIS4 staining at 2 hours and 6 hours post invasion, despite their ability to invade cells normally. If the PVM is established in conjunction with invasion, then the absence of UIS4 in the *pyrom1(-)* parasite vacuoles reflects a defect in the targeting of UIS4 to the PVM shortly after invasion and not necessarily a defect in establishing a PV.

The mechanisms by which UIS4 or other eTRAMPS are secreted and associate to the PVM are still unknown. A previously published analysis suggested eTRAMP proteins have a TMD with sequence similarity to the canonical rhomboid substrate, Spitz [32]. Here, we show for the first time that the transmembrane domain of an eTRAMP, PyUIS4, serves as a rhomboid substrate to DmRho-1. Unfortunately, we were not able to express a catalytically active pyROM1 (data not shown) to test activity against candidate substrates, including known substrates of the PyROM1 homolog, PfROM1 such as AMA1 or Spitz [39]. Therefore, we could not determine directly whether PyROM1 cleaves UIS4.

Little is known about the dynamic processes that occur just after invasion when the parasite has entered a new environment and must undergo dramatic changes. It is well established that cells sense changes in their environment from external cues that activate a signaling cascade through an internal sensor such as a surface receptor. A function in the activation of a signaling molecule has been described for rhomboid proteases in several species [80]. A recent study has attributed such a function to rhomboid protease activity in the parasite *Toxoplasma gondii* [81]. In this study, TgROM4 dominant negative mutants defective in replication were rescued by the over-expression of the cytoplasmic tail of TgAMA1 or PfAMA1 that resembled the rhomboid cleavage product [81]. Based on these results, intramembrane proteolysis of AMA1 is hypothesized to link the switch from invasion to replication. A biological role for rhomboid processing of AMA1 in *Plasmodium spp* has not been established since it is a minor event that is enhanced when the subtilisin protease PfSUB2, is inhibited [30], [38], [82]. Given that PfROM1 preferentially cleaved PfAMA1 relative to the TgROM4 ortholog, PfROM4 [39] and our current data, it is possible that ROM1 plays a role in a similar process in Plasmo*dium* species.

A separate study, using conditional knockdowns, showed TgROM4 functions as a sheddase of surface adhesins during gliding motility and invasion [83]. Our studies demonstrate that ROM1 does not directly function as a sheddase during cell entry, but instead is important in the stages just after invasion of hepatocytes. Therefore, as proposed for TgROM4 [81], we hypothesize that ROM1 activity links parasite invasion of host cells with parasite development within host cells. In a second model, ROM1 may function as a secretase that promotes the secretion of proteins such as UIS4 that are targeted to the PV or PVM for modification. A secretase activity may also serve to activate the secretion of a signal for parasite differentiation. In both models, ROM1 processing activity could occur prior to or after invasion. Further studies to identify substrates of ROM1 are needed to understand ROM1 function. Most importantly, this study provides new insights into the function of a rhomboid protease during intracellular development of Apicomplexan parasites.

## Materials and Methods

### Infection of mice, the parasite life cycle, mosquitoes, and cell lines

For routine passage of blood stage *Plasmodium yoelii* 17XNL parasites and for mosquito feedings, Swiss Webster mice (female, 4–5 weeks) were used. Mice were infected by either intraperitoneal or intravenous injections. For parasite infectivity assays such as liver parasite burden or blood stage infectivity, BALB/c mice (female, 6 weeks of age) were used. Blood stage parasites were harvested by intraocular bleeding of infected mice. All animals were purchased from either Charles River or Taconic.

Handling of mice and rodent malaria infections were conducted in accordance to approved protocols by the institutional animal use committees at Albert Einstein College of Medicine (protocol 20081001) and New York University School of Medicine (protocol 090809-02), in facilities approved by the Association for Assessment and Accreditation of Laboratory Animals. Protocols followed the recommendations of the Guide for the Care and Use of Laboratory Animals of National Institutes of Health Office of Laboratory Animal Welfare.

*Anopheles stephensi* mosquitoes were reared at 27°C and 80% humidity under a 12/12 h light/dark cycle, and adults were fed on 20% sucrose solution. Three to five- day old mosquitoes were used for feeding on infected mice with *P. yoelii 17XNL* and maintained at 24°C, 80% humidity.

All cells used were grown in Dulbecco's modified Eagle's medium supplemented with 10% heat inactivated fetal bovine serum, 1 mM Glutamine, 100 U Penicillin/ml, 10 µg/ml streptomycin, and maintained at 37°C and 5% CO_2_.

### Cloning of *pyrom1*

The gene encoding *pyrom1* was cloned by RT-PCR using total RNA from *Plasmodium yoelii* 17XNL mixed blood stage parasites extracted with TRIzol reagent (Invitrogen) followed by DNAse treatment (Ambion). Full length sequence of the *pyrom1* cDNA was amplified via 5′ and 3′ Rapid Amplification of cDNA Ends (SMARTRACE, Ambion BD Biosciences) using primers pyR1RACE5′R and pyR1RACE3′F. A touchdown PCR was carried out to increase the specificity of the PCR reaction. The PCR reaction products were separated by agarose gel and PCR products of various sizes were gel extracted (Qiagen) and cloned to a Topo vector (Invitrogen) for sequencing. Gene sequence of the open reading frame was further confirmed via RT-PCR using primers, pyR1.1-F and pyR1.1-R, specific to the deduced 5′ and 3′ ends of the *pyrom1* open reading frame. Exon/intron boundaries were confirmed to follow the conserved splicing motifs. Functional prediction of the translated amino acid sequence was obtained by using the Translate tool on the Expasy website (www.expasy.org). Prediction of transmembrane domains was performed using the TMHMM Server v. 2.0 (http://www.cbs.dtu.dk/services/TMHMM-2.0/) and HMMTOP (http://www.enzim.hu/hmmtop/). Primer sequences are listed in Table S3.

### RNA extraction and expression analysis by real-time qRT-PCR

Total RNA was prepared from synchronized *Plasmodium yoelii* parasites at various time points during development. Erythrocytic stages were synchronized by injecting 1×10^8^ purified schizonts into 5 wk old Swiss Webster mice (Charles River) by tail intravenous injection [84]. Specific synchronized erythrocytic stages were confirmed by Giemsa stained blood smears and collected at specified time points to obtain a ring stage, trophozoite stage, and schizont stage parasites. Mice infected with synchronized parasites were fully bled into heparinized complete medium. Infected blood was saponin lysed, centrifuged at 4°C, and the resulting parasite pellet was resuspended in 500 µl of TRIzol reagent (Invitrogen). To collect RNA from mosquito stage sporozoites, midguts or salivary glands were dissected from 100 infected (*P. yoelii* 17XNL) *A. stephensi* mosquitoes at 10 days post blood meal (midgut sporozoites) or 14 days post blood meal (salivary gland sporozoites). Midguts or salivary glands were dounce-homogenized in 500 µl of TRIzol reagent. For all of the TRIzol samples, RNA was extracted followed by treatment with DNAseI (Ambion), and passed through an RNeasy Cleanup column (Qiagen). Total RNA (1 µg) was incubated with random hexamers and used to make cDNA using the Superscript III First-Strand Synthesis System (Invitrogen) according to manufacturer instructions. Real-time PCR was carried out in a 10 µl volume using SYBR Green PCR master Mix (Applied Biosystems) and 1 µM gene specific primers. Real-time PCR was performed using the ABI Prism 7300 qPCR machine (Applied Biosystems). Transcript expression of *pyrom1* was normalized to the expression of the control gene, *18s* rRNA. The normalized expression for each gene was determined by the ddCt method [85] and values are expressed relative to the expression of the schizont stage. Primers used for real time PCR were designed using Primer Express Software (Table S3).

### Targeting vector construction and parasite transfection

*P. yoelii* 17XNL genomic DNA was used to amplify two 600 base pair (bp) fragments that are upstream and downstream of the *pyrom1* coding region using primers, pyR1.5′KpnI-F, pyR1.5′XhoI-R, pyR1.3′BamHI-F, pyR1.3′NotI-R, containing appropriate restriction sites to facilitate cloning of the PCR fragments into the PMD205GFP vector [48]. This vector contains a selection cassette that expresses the *P. berghei* dihydrofolate reductase-thymidylate synthase (pbDHFR-TS) gene fused to the Green Fluorescent Protein (GFP) open reading frame under the control of the pbDHFR promoter. The resulting targeting construct contains the pbDHFR-TS/GFP selection cassette flanked by the upstream (5′ARM) and downstream (3′ARM) fragments from the *pyrom1* genetic locus. The targeting vector was linearized using restriction enzymes KpnI, NotI, and ScaI prior to transfection. For the transfection, a total of 15 µg of linearized vector DNA was mixed with 5×10^7^ schizonts resuspended in 100 ul of cytomix [86] and transferred to an AMAXA cuvette for electroporation using the AMAXA nucleofector [87]. Pyrimethamine (1 mg/kg) treatment in the drinking water was started 24 hours post transfection and maintained until the appearance of drug resistant parasites.

### Southern blot analysis

Mixed blood stage parasites were released from host erythrocytes by treatment with 0.05% Saponin in ice-cold PBS. Parasite pellet was incubated with 2 mg/ml Proteinase K (Roche) at 37 C for 2 hours and genomic DNA (gDNA) was extracted using phenol/chloroform, chloroform extraction, followed by ethanol precipitation. Genomic DNA (2 µg) was treated with the restriction enzymes (New England Biolabs) overnight for complete digestion. To test for successful gene replacement in R1KO, gDNA was digested with enzymes ScaI/MscI or ScaI alone. To analyze integration of the N-terminal HA tag in R1HA parasite line, gDNA was digested with enzymes BstZ171 and MscI. Restricted DNA was separated by gel electrophoresis on a 0.8% agarose gel, transferred via capillary action to a Nylon membrane (Roche). DNA probe specific to the 3′homologous region was amplified using PCR and Digoxigenin (DIG) labeled nucleotides (Roche). Probe hybridization and chemiluminescent detection were carried out using manufacturer's instructions (Roche).

### N-terminal tagging of *pyrom1* with a triple HA epitope

The spliced open reading frame amplified from *P. yoelii* 17XNL cDNA was cloned into a vector containing an N-terminal triple HA tag using the EcoRI and NotI restriction sites [33]. The 5′ flanking region (FR) was amplified from gDNA (1.5 kb) and was cloned into a pBluescript SK(-) (Stratagene) plasmid using restriction sites ApaI and XbaI to generate pB5′FR. The HA-pyrom1 fragment was subsequently cloned within the XbaI and Not I sites to generate the pB5′FRHAR1 plasmid. The 3′FR (1 kb) was then cloned using NotI and SacII sites into the pB5′HAR1 plasmid to generate pB5′FRHAR13′FR plasmid. The entire insert containing a 5′FR, an N terminally tagged *pyrom1* ORF, and a 3′FR, was then cut out with ApaI and SacII and was cloned into the PL0006 vector that contains the hDHFR selectable marker (Leiden) to ultimately generate the pR1HA knock-in vector. Prior to *P. yoelii* transfection the pR1HA vector was linearized with the restriction enzyme Aria in order to promote homologous recombination within the 5′FR. Transfection was carried out as described above. Following pyrimethamine treatment (1 mg/kg), recombinant parasites were analyzed by PCR and Southern blot analysis of gDNA. Expression of tagged ROM1 was verified by Western Blot. Immunofluorescence microscopy was then carried out to analyze expression and localization of pyROM1 during different stages of the parasite life cycle. Primer sequences are listed in Table S3.

### Immunofluorescence microscopy of pyROM1-HA

Thin smears of mixed blood stages were fixed in ice cold acetone/methanol (1∶1) for 3 minutes, blocked with 3% BSA/PBS, incubated with appropriate primary antibody, followed by secondary antibody. Washes were carried out using PBS-Tween 20 (0.005%). Salivary gland sporozoites were centrifuged onto 8-well glass Labtek chambers (Nunc), fixed with 4% Paraformaldehyde, and permeabilized with 0.2% TritonX-100. Fixed sporozoites were then blocked and incubated with antibody as described above. Washes were carried out using 1× PBS. For IFA of liver stages, infected Hepa1–6 cells at specified time points were fixed with ice cold methanol for 15 minutes and incubated with antibodies as described below. To capture invading R1HA sporozoites, Hepa1–6 cells were infected for 10 minutes, fixed with 4% paraformaldehyde, blocked, and stained with α-pyCSP to detect extracellular or invading parasites, permeabilized with ice cold 100% methanol, and incubated with rabbit α-HA to detect pyROM1-HA. Primary antibodies used were 1/1000 rabbit α-HA (Sigma), 1/100 α-pyCSP 2F6 from hybridoma supernatants, 1/500 α-HA rat mAb 3F10 (Roche), 1/1000 rabbit α-pyMAEBL YM2T8 antisera [88]–[89], 10 ug/ml rabbit α-PfBiP antisera (MR4 ATCC, MRA-20), 1/500 and rabbit αUIS4 antisera [15]. Secondary antibodies conjugated to Alexa 488 or Alexa 568 (Molecular Probes) were used and 0.02 µg/ml of 4′6-diamidino-2-phenylindole (DAPI) was used for nuclear staining. Images were taken at 100× magnification with the Upright Olympus BX61 microscope using the IP Lab 4.0.8 software through the Analytical Imaging Facility of the Albert Einstein College of Medicine.

### Phenotypic analysis of intra-erythrocytic parasite infectivity in mice

An inoculum of 1×10^4^ mixed blood stage parasites from R1KO and wildtype parasites was injected intravenously into five 6-week old female BALB/c mice (Charles River). Parasitemia was monitored by daily blood smears from each infected mouse, stained with Giemsa (Sigma) and counted under light microscopy. Percent parasitemia was determined by counting at least 1,000 red blood cells per smear and calculated as the percentage of infected red blood cells (iRBC) to total number of red blood cells.

### Phenotypic analysis of parasite development in mosquitoes

Swiss Webster mice (4 weeks, female) were injected intravenously with 5×10^7^ infected RBC from either wildtype or R1KO parasites. Two days later, starved *Anopheles stephensi* mosquitoes were fed on the infected mice harboring mature gametocytes for 15 minutes. A second 5 minute feeding was carried out the following day. Parasite transmission and infectivity was determined by counting the number of oocysts from dissected midguts at day 8 post feeding and the number of salivary gland associated sporozoites at day 14 post feeding under a light microscope.

### Gliding motility assay

At day 14 post blood meal feeding, mosquito salivary glands were dissected and sporozoites were collected and counted using a hemocytometer. Salivary gland sporozoites (3×10^4^) were added to 8-well Lab-Tek glass slides (Nalgene) that had been pre-coated with 200 µl of 10 ug/ml α-pyCSP (mAb 2F6) antibody in PBS at room temperature, overnight. Loaded Lab-Tek slides were incubated for 1 hr at 37°C to allow sporozoite gliding, after a brief centrifugation (500 RPM, 1 minute). Media was removed and wells were immediately fixed with 4% Paraformaldehyde at 4°C overnight. Wells were blocked with 1% BSA/PBS solution, incubated with biotinylated mAb 2F6 (1 hour, 37°C, 1/100 dilution), and stained with streptavidin-Alexa 488 (Molecular Probes). Trails were visualized and counted under a fluorescence microscope.

### Cell traversal assay

Salivary gland sporozoites (3×10^4^) were dissected in 1% BSA/DMEM and loaded onto Hepa 1–6 cell monolayers in the presence of 1 mg/ml FITC-dextran (10,000 MW; Molecular Probes). In control wells, sporozoites were pre-treated for 10 minutes with 1 mM of Cytochalasin D on ice before being loaded onto cells. Sporozoites were centrifuged (1000 RPM, 3 minutes) and incubated for 1 hour at 37°C. Cells were washed extensively with 1×PBS to remove excess FITC-dextran that had not been taken up and were fixed with 4% Paraformaldehyde, mounted and visualized under a fluorescent microscope at 40× magnification. The number of FITC-positive cells was counted in at least 30 fields and values represented are FITC-positive cells per field.

### Sporozoite invasion and development assays

For invasion assay, semi-confluent Hepa 1–6 cells were loaded with 5×10^4^ *P. yoelii* WT or R1KO salivary gland sporozoites and centrifuged (1000RPM, 3 minutes). After 2 hour or 6 hour incubation at 37°C to allow invasion, media was removed and cells were washed with PBS, fixed with 4% paraformaldehyde for 30 minutes, and blocked for 1 hour at 37°C with 1% BSA/PBS. Cells were incubated with the α-pyCSP 2F6 mAb following the Red-Green double staining method to distinguish intracellular versus extracellular parasites [60]–[61]. For liver development assays, Hepa 1–6 cells were loaded with 5×10^4^ *P. yoelii* Wt or R1KO salivary gland sporozoites, spun down and incubated for 6, 12, and 24 hours to allow EEF development. At the end of each time point, cells were fixed with ice-cold methanol and stained with either mAb 2F6 (α-pyCSP) or mAb 2E6 (α-HSP70) [90] to visualize and quantify parasite development. At least 50 fields were counted per well and each experiment was done in duplicate or triplicate. For detection of UIS4 positive PVM salivary gland sporozoites were incubated for 2 or 6 hours with Hepa 1–6 cells as describe above. After each time point, cells were fixed with 4% paraformaldehyde for 30 minutes at room temperature followed by permeabilization with 100% ice cold methanol for 10 minutes. Double staining was performed using α-PyCSP 2F6 mouse monoclonal antibody and α-PyUIS4 rabbit polyclonal antibody for 1 hour at 37°C. Staining with secondary antibodies was followed with goat α-mouse IgG–Alexa 488 and goat α-rabbit IgG-Alexa 568. The percent of parasites double staining (CSP-UIS4) over total number of parasites (CSP only) was recorded in duplicate. Statistical analysis was carried out using either One-way ANOVA multiple comparison test or unpaired t-test with the GraphPad Prism software.

### Quantitative analysis of liver infectivity *in vivo*

Analysis of malaria infection in mouse livers was carried out as described previously [50]. Salivary gland sporozoites from wildtype or R1KO infected mosquitoes were collected and counted using a hemocytometer. Female BALB/c mice 6 weeks of age (purchased from Charles River) were intravenously injected with 1000 or 10,000 sporozoites. Four or five mice were independently injected per parasite line. Livers were harvested 36 hours later and homogenized in 10 ml of TRIzol reagent (Invitrogen). RNA was extracted from 1.5 ml of liver homogenate, treated with DNAseI (Ambion) and purified with an RNeasy purification column (Qiagen). Total RNA (4 µg) was used to make cDNA using the Superscript III cDNA synthesis system (Invitrogen) and random hexamers as primers. Quantitative PCR was carried out with the ABI 7300 apparatus using the POWER SYBR green master mix (Applied Biosystems) in a 20 µl reaction volume containing 2 µl of cDNA and 1 µM of primers. Test Primers to detect parasite burden within liver were to the *Plasmodium* 18s rRNA gene and the internal control primers were specific to the mouse GAPDH gene. Relative transcript quantification was determined using the 2^−ddCt^ method.

### Heterologous cleavage assay in COS7 cells

HA-tagged rhomboid expressing plasmids (DmRho-1, TgROM5, TgROM5^SA^) were obtained from Dr. Sinisa Urban as published in [33]. A truncated PyUIS4 that excludes the predicted signal sequence and most of the C-terminal domain was amplified from sporozoite cDNA. The reverse primer was designed to include a 2xMyc tag. This PCR product was fused to GFP preceded by an IgK leader sequence via PCR joining using primers listed in Table S3. The resulting PCR was inserted into the pcDNA3.3 TOPO TA cloning vector. The template for GFP is from the Spitz-GFP expression plasmid used in [33]. The cleavage assay was carried out as described in [33], [39] with minor modifications. Briefly, COS7 were seeded on 6-well plates and transfected with plasmid DNA for transient expression using Fugene6 (Roche) reagent following manufacturer's protocol. For PyUIS4TMGFP, 250 ng of plasmid DNA was used per well and 100 ng of plasmid DNA was used for rhomboid constructs. A pBluescript (Stratagene) plasmid was used as filler DNA. At 18 hours post-transfection, media was removed, cells were washed with serum-free media (SFM), and 800 µl of SFM containing protease inhibitor Galardin (GM_600, Biomol) was added. The conditioned media was collected and cells were harvested by lysis with sample buffer at 18–24 hours later. Media fraction was concentrated using Centricon (Millipore) centrifugal concentrator with a cut off of 3 kDa. Western blot analysis was carried out for both media fractions and cell lysates. Anti-GFP antibody (Santa Cruz Biologicals) was used to detect PyUIS4TMGFP and anti-HA mAb clone 3F10 (Roche) was used to detect rhomboid constructs.

### Electron microscopy of early hepatic stages

Salivary gland sporozoites (1×10^6^) were dissected and loaded onto a confluent monolayer of Hepa1–6 cells at an MOI of 2∶1 (5×10^5^), centrifuged (1000 RPM, 3 minutes), and allowed to invade for two hours. Infected cells were washed with media containing 10× Pen/Strep and 0.25 µg/ml of Fungizone (Invitrogen) to remove debris and unbound sporozoites. Development was allowed to continue another two hours for a total of four hours of development. Cells were then washed with PBS, trypsinized, and washed one more time with PBS, followed by fixation with 2% Paraformaldehyde/2.5% Glutaraldehyde for 1 hour at room temperature. Cells were washed with PBS and postfixed in 1% osmium tetroxide (Polysciences Inc., Warrington, PA) for 1 hour. Samples were then rinsed extensively in dH20 prior to en bloc staining with 1% aqueous uranyl acetate (Ted Pella Inc., Redding, CA) for 1 hour. Following several rinses in dH20, samples were dehydrated in a graded series of ethanol and embedded in Eponate 12 resin (Ted Pella Inc.). Sections of 95 nm were cut with a Leica Ultracut UCT ultramicrotome (Leica Microsystems Inc., Bannockburn, IL), stained with uranyl acetate and lead citrate, and viewed on a JEOL 1200 EX transmission electron microscope (JEOL USA Inc., Peabody, MA). Image J software was used for quantification of the PVM/PV area in the EM images and statistical analysis was carried out with the GraphPad Prism software using unpaired t test. Measurements were made by on blinded images.

## Supporting Information

Figure S1 **Schematic representation of pyROM1 gene structure and sequence.** Sequence of pyROM1 was obtained from blood stage cDNA using 5′RACE and 3′RACE. A) The gene comprises two predicted open reading frames on PlasmoDB (py00728 and py00729). *Pyrom1* is composed of four exons (including UTRs) and three introns. Exons (labeled with roman numerals I–IV) are represented by the gray boxes and the introns by the thin gray lines. Numbers below each exon represents the nucleotide length of the coding region of the exon. The codons for the catalytic Serine (S) and Histidine (H) residues are encoded within exon III. B) Nucleotide sequence of the Open Reading Frame (ORF) of *pyrom1* with the corresponding translated amino acid sequence. Nucleotide or amino acid position is marked by the numbers on the left margin. The microneme targeting motif within the N-terminal tail of pyROM1 is boxed. The transmembrane domains are shaded in gray. The catalytic Serine (S) and Histidine (H) are in red color.
(TIF)

Figure S2 **Gene expression controls for qRT-PCR in various stages of the** ***Plasmodium yoelii*** **life cycle.** Quantitative RT-PCR (qRT-PCR) was carried out using the method described in this work using the same cDNA used to obtain results in Figure 1A. Primers for qRT-PCR were designed to amplify PyADA, PyAMA, PyTUB1, PyUIS3, and PyCSP. Transcript expression was normalized to the expression levels of the ribosomal rRNA 18s. The calibrator used to plot relative expression varies, and is denoted as a subscript in the title of the legend on the Y-axis. (A) Expression analysis of three gene expressed during erythrocytic blood stages. PyADA encodes the enzyme Adenosine Deaminase involved in purine salvage metabolism. PyTUB1 encodes for alpha tubulin1, a structural protein prominent in the zoite stages. PyAMA (PlasmoDB: Py01581) is a gene whose product is a microneme invasion adhesin involved in host cell invasion expressed in erythrocytic and sporozoite stages. (B) Expression analysis of three genes expressed during mosquito and pre-erythrocytic stages. PyCSP (PlasmoDB: Py03168) encodes for Circumsporoozite protein expressed during oocyst development, salivary gland sporozoite, and early pre-erythrocytic stages. PyUIS3 (PlasmoDB: Py03011) encodes for Upregulated in Sporozoite 3, a member of the eTRAMP family specifically upregulated in salivary gland sporozoites with continued expression during pre-erythrocytic stages.
(TIF)

Figure S3 **Genetic disruption of the** ***pyrom1*** **gene.** A) Schematic of strategy for disrupting the *pyrom1* locus using integration plasmid R1INT. A 600 bp fragment representing the middle portion of the *pyrom1* ORF excluding the 5′ and 3′ ends was amplified from gDNA by PCR. This fragment was cloned onto the PMD205GFP vector [48] using BamHI and NotI restriction sites to generate R1INTPMD205GFP targeting vector. The plasmid was linearized using restriction enzyme BsiWI to facilitate homologous recombination. B) For Southern blot analysis, 3 µg of genomic DNA was digested with restriction enzyme PacI (New England Biolabs). Digested DNA was separated on a 0.8% agarose gel and transferred to a nylon membrane (Roche Applied Science). A probe (orange line) encompassing the region used for homologous recombination for the *pyrom1* gene was amplified by PCR using digoxigenin (DIG)-labeled UTP nucleotides (Roche Applied Science). Southern blot was revealed using an anti-DIG antibody coupled to peroxidase (Roche Applied Science). A unique specific band at 1858 bp (black arrowhead) for the wildtype gDNA is seen in one of the transfected clones (lane 2) and two bands at 7565 bp and 2343 bp (pink arrowheads) representing successful disruption of *pyrom1* are seen in two clones (lanes 1 and 3). As a control, the PacI linearized episome (Epi) runs at the expected size of ∼8775 bp (black arrow). C) Verification of successful *pyrom1* disruption at the RNA level. Two different primer sets (pyrom1.1 and pyrom1.2) were used to amplify the *pyrom1* gene from cDNA of wildtype parasites and disrupted parasites clone 1 (lane 1 from southern blot in B). Amplification of ADA, an unrelated gene, was used as an internal control. No Reverse Transcriptase (RT) cDNA samples were used as a control for gDNA contamination.
(TIF)

Figure S4 **Representative electron microscopy images of** ***pyrom1(-)*** **parasites displaying the parasitophorous vacuole phenotype.** Intracellular parasites at 4 hour development within Hepa 1–6 host cells. Notice the well defined white ‘halo’ surrounding the parasite within the PV observed in the wildtype parasites (A,C,D,F) in contrast to phenotype displayed by R1KO developing parasites (B,D,F,G). Inset shows a close up of the boundary between the parasite and the host cell cytoplasm. Scale bars are equal to 0.5 µm. Abbreviations: PVM- Parasitophorous vacuole membrane, PV-Parasitophorous vacuole.
(TIF)

Table S1 ***In vivo*** **transmission and pre-patent period of** ***pyrom1 (-)*** **parasites.** Balb/c mice (Female 6 weeks, N = 5) were infected by either natural mosquito bite (10 mosquitoes/mouse for 5 mins) or by intravenous injection of salivary gland sporozoites (20, 200, 2000). Peripheral blood parasitemia was monitored by Giemsa stained blood smears starting at day 3. Pre-patent period represents the number of days from initial infection until the appearance of blood parasites.
(DOC)

Table S2 **Quantitative analysis of the parasitophorous vacuole space in** ***pyrom1(-)*** **parasites.** Electron microscopy images of intracellular parasites at four hours development within hepatocytes were used to quantify the area of the PV space and qualitatively describe the parasitophorous vacuole. Observers were blinded as to image identity. Statistical analysis was performed with GraphPad Prism software using unpaired t test (p value = 0.0005).
(DOC)

Table S3 **Primer sequences used in study.** List of primers used throughout the study as referred to in the materials and methods. Sequences underlined correspond to restriction enzyme sites used to facilitate cloning. Bold letters within primer nucleic acid sequence correspond to the sequence of PyUIS4 sequence used to amplify the transmembrane domain (PlasmoDB accession number py00204).
(DOC)
