# Supplementary figures and images for: Plasmodium Protease ROM1 Is Important for Proper Formation of the Parasitophorous Vacuole
Source: PLoS Pathog. 2011 Sep 1;7(9):e1002197. doi: 10.1371/journal.ppat.1002197 (PMC3164628; doi:10.1371/journal.ppat.1002197)

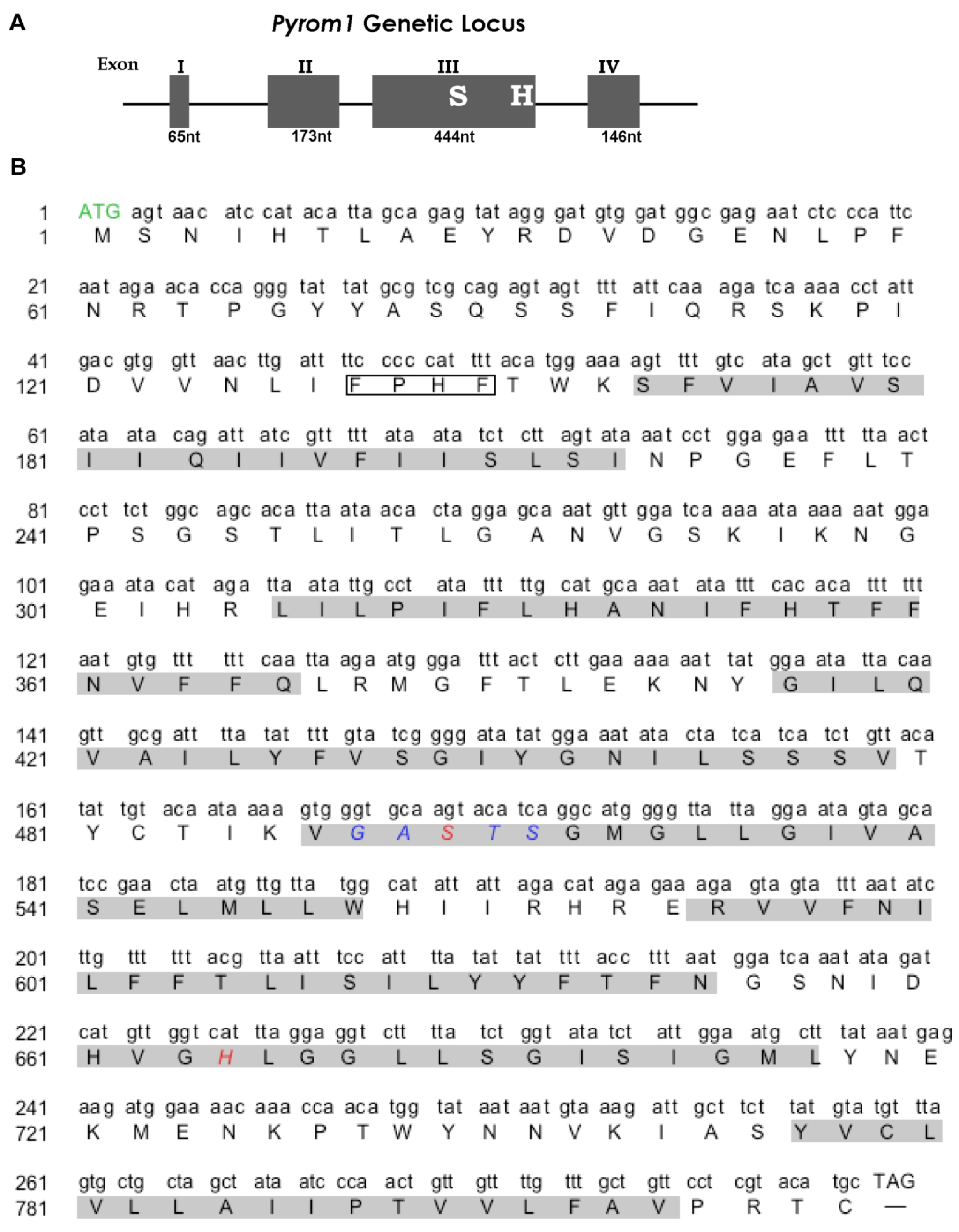

Supplement: Figure S1 — Schematic representation of pyROM1 gene structure and sequence. Sequence of pyROM1 was obtained from blood stage cDNA using 5′RACE and 3′RACE. A) The gene comprises two predicted open reading frames on PlasmoDB (py00728 and py00729). Pyrom1 is composed of four exons (including UTRs) and three introns. Exons (labeled with roman numerals I–IV) are represented by the gray boxes and the introns by the thin gray lines. Numbers below each exon represents the nucleotide length of the coding region of the exon. The codons for the catalytic Serine (S) and Histidine (H) residues are encoded within exon III. B) Nucleotide sequence of the Open Reading Frame (ORF) of pyrom1 with the corresponding translated amino acid sequence. Nucleotide or amino acid position is marked by the numbers on the left margin. The microneme targeting motif within the N-terminal tail of pyROM1 is boxed. The transmembrane domains are shaded in gray. The catalytic Serine (S) and Histidine (H) are in red color. (TIF) [file ppat.1002197.s001.tif]

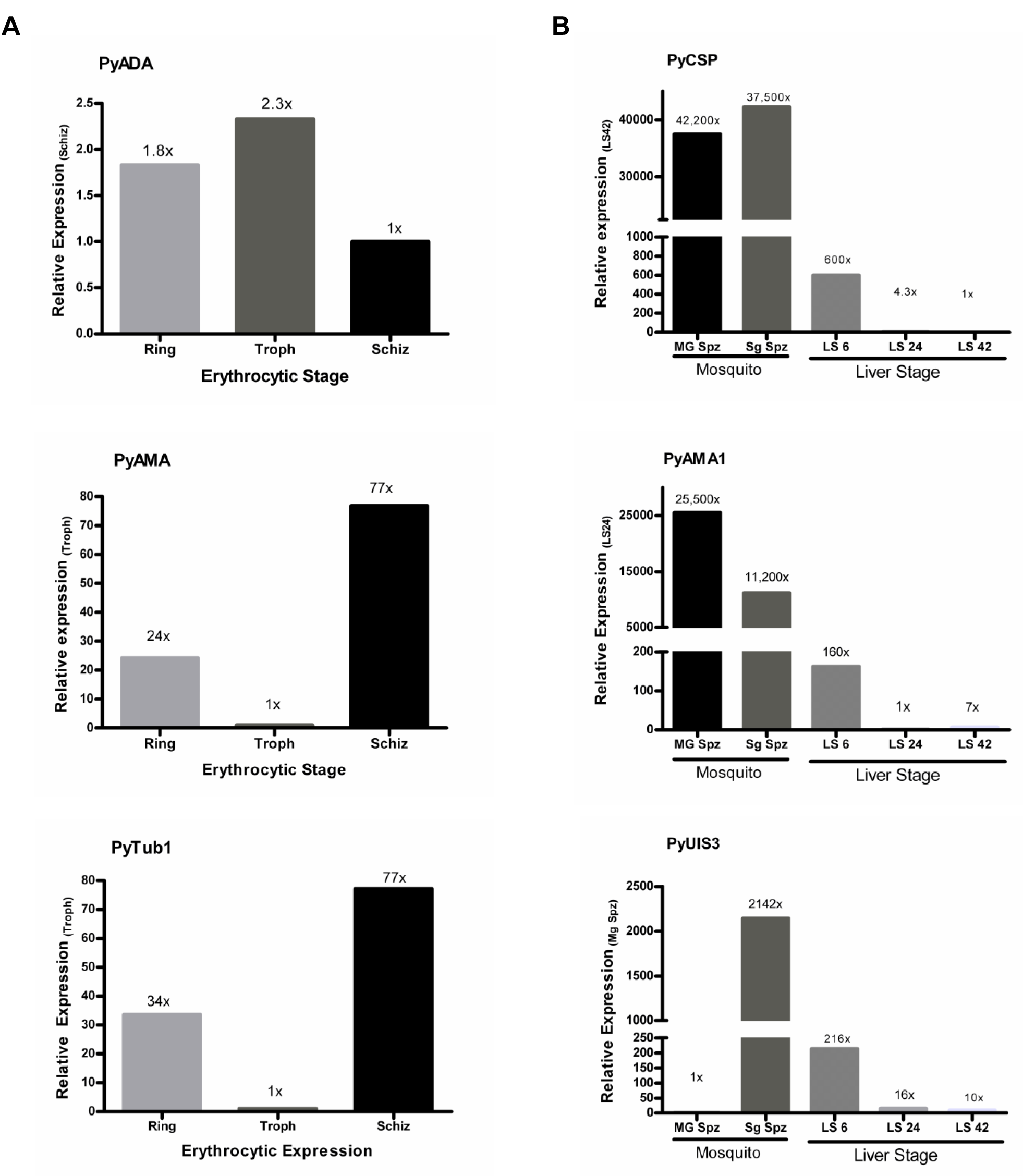

Supplement: Figure S2 — Gene expression controls for qRT-PCR in various stages of the Plasmodium yoelii life cycle. Quantitative RT-PCR (qRT-PCR) was carried out using the method described in this work using the same cDNA used to obtain results in Figure 1A. Primers for qRT-PCR were designed to amplify PyADA, PyAMA, PyTUB1, PyUIS3, and PyCSP. Transcript expression was normalized to the expression levels of the ribosomal rRNA 18s. The calibrator used to plot relative expression varies, and is denoted as a subscript in the title of the legend on the Y-axis. (A) Expression analysis of three gene expressed during erythrocytic blood stages. PyADA encodes the enzyme Adenosine Deaminase involved in purine salvage metabolism. PyTUB1 encodes for alpha tubulin1, a structural protein prominent in the zoite stages. PyAMA (PlasmoDB: Py01581) is a gene whose product is a microneme invasion adhesin involved in host cell invasion expressed in erythrocytic and sporozoite stages. (B) Expression analysis of three genes expressed during mosquito and pre-erythrocytic stages. PyCSP (PlasmoDB: Py03168) encodes for Circumsporoozite protein expressed during oocyst development, salivary gland sporozoite, and early pre-erythrocytic stages. PyUIS3 (PlasmoDB: Py03011) encodes for Upregulated in Sporozoite 3, a member of the eTRAMP family specifically upregulated in salivary gland sporozoites with continued expression during pre-erythrocytic stages. (TIF) [file ppat.1002197.s002.tif]

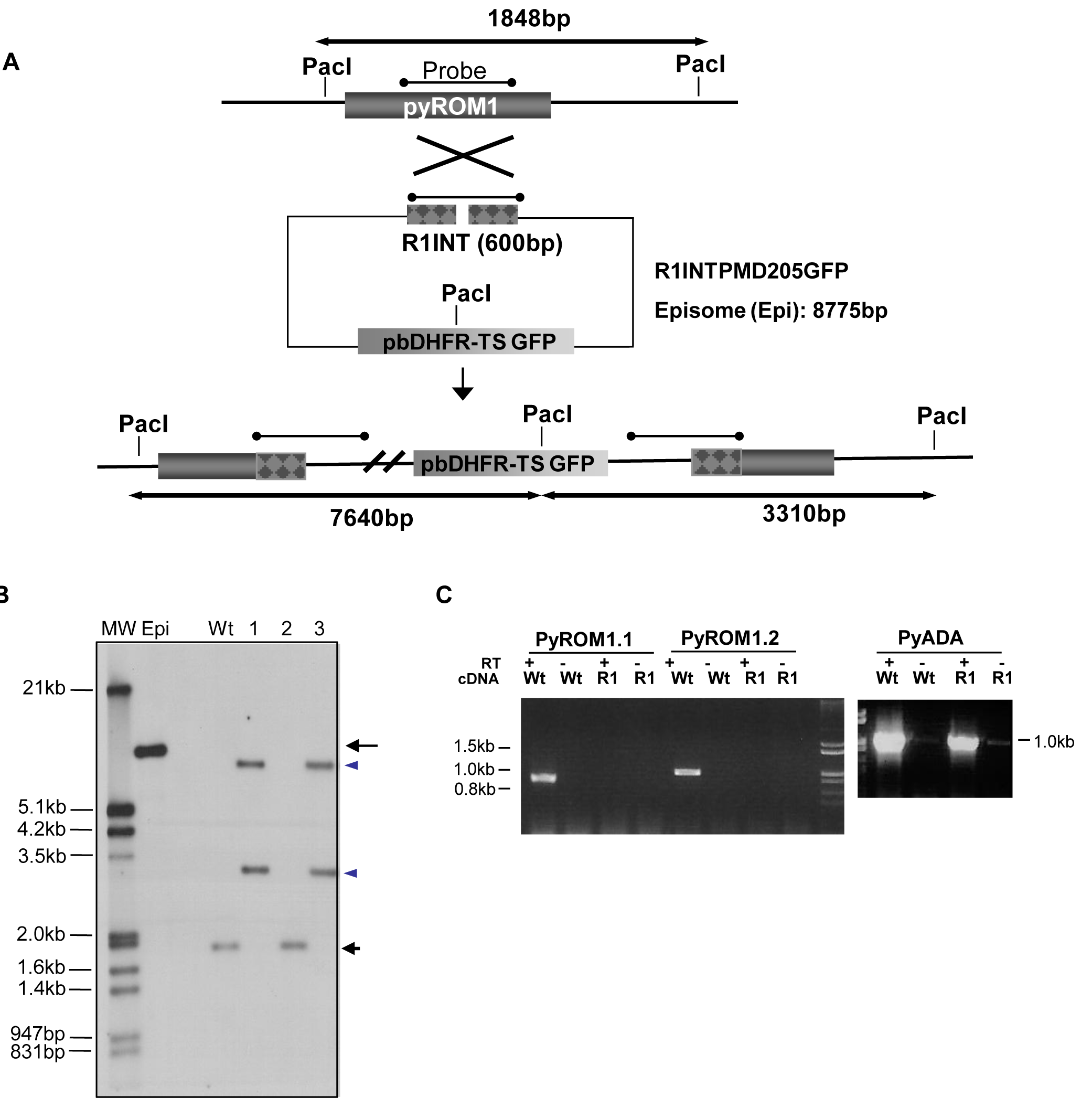

Supplement: Figure S3 — Genetic disruption of the pyrom1 gene. A) Schematic of strategy for disrupting the pyrom1 locus using integration plasmid R1INT. A 600 bp fragment representing the middle portion of the pyrom1 ORF excluding the 5′ and 3′ ends was amplified from gDNA by PCR. This fragment was cloned onto the PMD205GFP vector [48] using BamHI and NotI restriction sites to generate R1INTPMD205GFP targeting vector. The plasmid was linearized using restriction enzyme BsiWI to facilitate homologous recombination. B) For Southern blot analysis, 3 µg of genomic DNA was digested with restriction enzyme PacI (New England Biolabs). Digested DNA was separated on a 0.8% agarose gel and transferred to a nylon membrane (Roche Applied Science). A probe (orange line) encompassing the region used for homologous recombination for the pyrom1 gene was amplified by PCR using digoxigenin (DIG)-labeled UTP nucleotides (Roche Applied Science). Southern blot was revealed using an anti-DIG antibody coupled to peroxidase (Roche Applied Science). A unique specific band at 1858 bp (black arrowhead) for the wildtype gDNA is seen in one of the transfected clones (lane 2) and two bands at 7565 bp and 2343 bp (pink arrowheads) representing successful disruption of pyrom1 are seen in two clones (lanes 1 and 3). As a control, the PacI linearized episome (Epi) runs at the expected size of ∼8775 bp (black arrow). C) Verification of successful pyrom1 disruption at the RNA level. Two different primer sets (pyrom1.1 and pyrom1.2) were used to amplify the pyrom1 gene from cDNA of wildtype parasites and disrupted parasites clone 1 (lane 1 from southern blot in B). Amplification of ADA, an unrelated gene, was used as an internal control. No Reverse Transcriptase (RT) cDNA samples were used as a control for gDNA contamination. (TIF) [file ppat.1002197.s003.tif]

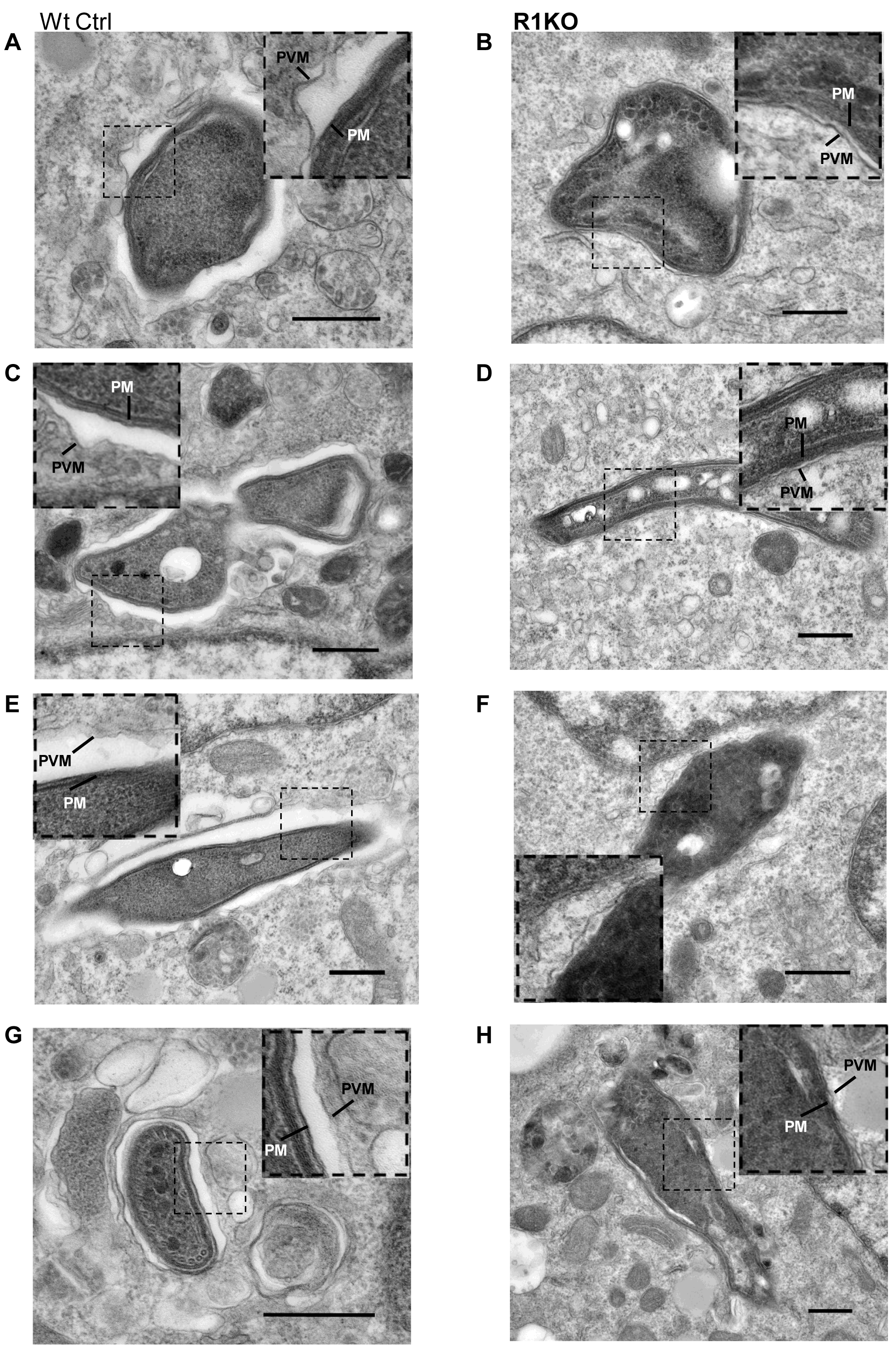

Supplement: Figure S4 — Representative electron microscopy images of pyrom1(-) parasites displaying the parasitophorous vacuole phenotype. Intracellular parasites at 4 hour development within Hepa 1–6 host cells. Notice the well defined white ‘halo’ surrounding the parasite within the PV observed in the wildtype parasites (A,C,D,F) in contrast to phenotype displayed by R1KO developing parasites (B,D,F,G). Inset shows a close up of the boundary between the parasite and the host cell cytoplasm. Scale bars are equal to 0.5 µm. Abbreviations: PVM- Parasitophorous vacuole membrane, PV-Parasitophorous vacuole. (TIF) [file ppat.1002197.s004.tif]
