# Supplementary material for: Plasmodium Protease ROM1 Is Important for Proper Formation of the Parasitophorous Vacuole
Source: PLoS Pathog. 2011 Sep 1;7(9):e1002197. doi: 10.1371/journal.ppat.1002197 (PMC3164628; doi:10.1371/journal.ppat.1002197)
Supplement: Table S1 — In vivo transmission and pre-patent period of pyrom1 (-) parasites. Balb/c mice (Female 6 weeks, N = 5) were infected by either natural mosquito bite (10 mosquitoes/mouse for 5 mins) or by intravenous injection of salivary gland sporozoites (20, 200, 2000). Peripheral blood parasitemia was monitored by Giemsa stained blood smears starting at day 3. Pre-patent period represents the number of days from initial infection until the appearance of blood parasites. (DOC) [file ppat.1002197.s005.doc]

| **Table S1. Prepatency of *pyrom1(-)* sporozoite transmission** | | | | |
| --- | --- | --- | --- | --- |
| **Infection by Mosquito Bite^a^** | |  |  |  |
|  | **Parasite Population** | **No. inoculated/ No. infected^b^** | **Pre-patent period^c^** |  |
| Experiment 1 | Wt Ctrl | 5 of 5 | Day 3 |  |
|  | R1INT | 5 of 5 | Day 3 |  |
| Experiment 2 | Wt Ctrl | 5 of 5 | Day 3 |  |
|  | R1KO-2 | 5 of 5 | Day 3 |  |
| **Infection by Intravenous (IV) Injection** | | |  |  |
| **Spz/ mouse^d^** | **Parasite Population** | **No. inoculated/ No. infected^b^** | **Pre-patent period^c^** |  |
| 20 | Wt Ctrl | 5 of 5 | Day 3 |  |
|  | R1KO-2 | 5 of 5 | Day 3 |  |
| 200 | Wt Ctrl | 5 of 5 | Day 3 |  |
|  | R1KO-2 | 5 of 5 | Day 3 |  |
| 2000 | Wt Ctrl | 5 of 5 | Day 3 |  |
|  | R1KO-2 | 5 of 5 | Day 3 |  |
| a) Mice were infected by the bite of mosquitoes (10 mosquitoes/mouse). | | | |  |
| b) Number of mice inoculated/number of mice with blood parasitemia. | | | |  |
| c) Number of days from inoculation until detection of blood parasitemia. | | | |  |
| d) Number of salivary gland sporozoites (spz) inoculated by tail IV injection. | | | |  |
