# Supplementary material for: Plasmodium Protease ROM1 Is Important for Proper Formation of the Parasitophorous Vacuole
Source: PLoS Pathog. 2011 Sep 1;7(9):e1002197. doi: 10.1371/journal.ppat.1002197 (PMC3164628; doi:10.1371/journal.ppat.1002197)
Supplement: Table S2 — Quantitative analysis of the parasitophorous vacuole space in pyrom1(-) parasites. Electron microscopy images of intracellular parasites at four hours development within hepatocytes were used to quantify the area of the PV space and qualitatively describe the parasitophorous vacuole. Observers were blinded as to image identity. Statistical analysis was performed with GraphPad Prism software using unpaired t test (p value = 0.0005). (DOC) [file ppat.1002197.s006.doc]

| **Table S2. Comparison of PV Characteristics** | | |
| --- | --- | --- |
| **Characteristic** | **Wt Ctrl** | **R1KO** |
| PV with Halo | 33 of 38 | 23 of 44 |
| Tight fitting PV | 1 of 38 | 19 of 42 |
| Cytoplasmic/ Nucleoplasmic | 4 of 38 | 2 of 42 |
| Mean Area Ratio (PV/Parasite) μM | 0.09 ± 0.01* | 0.017 ± 0.02 |
| * Statistically significant (P-value 0.0007) | |  |
