# Supplementary material for: Plasmodium Protease ROM1 Is Important for Proper Formation of the Parasitophorous Vacuole
Source: PLoS Pathog. 2011 Sep 1;7(9):e1002197. doi: 10.1371/journal.ppat.1002197 (PMC3164628; doi:10.1371/journal.ppat.1002197)
Supplement: Table S3 — Primer sequences used in study. List of primers used throughout the study as referred to in the materials and methods. Sequences underlined correspond to restriction enzyme sites used to facilitate cloning. Bold letters within primer nucleic acid sequence correspond to the sequence of PyUIS4 sequence used to amplify the transmembrane domain (PlasmoDB accession number py00204). (DOC) [file ppat.1002197.s007.doc]

| **Table S3. Primer sequences used in study** | | |  |  |  |
| --- | --- | --- | --- | --- | --- |
| **Cloning** |  |  |  |  |  |
| pyR1 5'RACE R | TCGCCATCCACATCCCTATACTCTGCTAATG | | |  |  |
| pyR1 3'RACE F | GCAGAGTATAGGGATGTGGATGGCGAGAATC | | | |  |
| PyR1.1 F | ATGAGTAACATCCATACATTAGCAGAGTAT | | |  |  |
| pyR1.1 R | CTAGCATGTACGAGGAACAGCA | | |  |  |
| **qRT-PCR** |  |  |  |  |  |
| pyR1qPCR F | ATGTTGGTCATTTAGGAGGTCTTTTATC | | |  |  |
| pyR1qPCR R | CATGTTGGTTTGTTTTCCATCTTCTC | | |  |  |
| 18srRNA F | GGGGATTGGTTTTGACGTTTTTGCG | | |  |  |
| 18srRNA R | AAGCATTAAATAAAGCGAATACATCCTTAT | | |  |  |
| **Targeting Vector** |  |  |  |  |  |
| pyR1.5'KpnI F | GGTACCTTAATTAAGCAAGCTTCCGAA | | |  |  |
| pyR1.5XhoI R | CTCGAGACAATGAAAAAGGAAGAAACACTC | | |  |  |
| pyR1.3'BamHI F | GGATCCAGATGGAAAACAAACCAACAT | | |  |  |
| pyR1.3'NotI R | GCGGCCGCTGACGCTTAGTGTTTAAATATTGTT | | | |  |
| GFP F | ATGAGTAAAGGAGAAGAACTTTTCACT | | |  |  |
| **pyR1HA Knock In Construct** | |  |  |  |  |
| pyR1.1EcoRI F | GAATTCATGAGTAACATCCATACATTAGCAGAGTAT | | | |  |
| pyR1.1NotI R | GCGGCCGCCTAGCATGTACGAGGAACAGCA | | |  |  |
| pyR1.5'ApaI F | GGGCCCGAGATACAAAATTATTGTTCACGC | | |  |  |
| pyR1.5'XbaI R | TCTAGATTTCTGTTATTAATTTTTTTTATCAATAA | | | |  |
| pyR1.3'NotI F | GCGGCCGCAAAGTTATAGTAAATATGCTTTGTTCC | | | |  |
| pyR1.3'SacII R | CCGCGGAATTCATATTTATTCCTATGTATATATGTTTG | | | |  |
| 3xHA F | ATGTACCCATACGATGTTCCT | |  |  |  |
| 3xHA R | ATAGCCCGCATAGTCAGGAACATC | | |  |  |
| **Internal Control Primers** | |  |  |  |  |
| pyAda F | ATGATGGAAATTCCAACTGAAGA | | |  |  |
| pyAda R | TTATTTGTATAGTTCATCCATGCCA | | |  |  |
| **UIS4TMGFP construction** | |  |  |  |  |
| PyUIS4_GFP F | GGA TGA ACT ATA CAA AGA ATT C**GA GAC AAA CGA TCC AAA GCC** | | | | |
| PyUIS4_2xMyc R | TTA ATT CAG ATC CTC TTC TGA GAT GAG TTT TTG TTC GGG GCC ATT CAG ATC CTC TTC TGA GAT GAG TTT TTG TTC GGA TCC **TCT AAT TCC AAA TTT TTC** | | | | |
